# Supplementary material for: cellGeometry: ultra-fast single-cell deconvolution of bulk RNA-Seq using a geometric solution
Source: Nat Commun. 2026 Jul 23;17:8995. doi: 10.1038/s41467-026-75762-7 (PMC13507090; doi:10.1038/s41467-026-75762-7)
Supplement: Supplementary file 3 [file 41467_2026_75762_MOESM3_ESM.pdf]

nature portfolio

# Peer Review File

# cellGeometry: ultra-fast single-cell deconvolution of bulk RNA-Seq using a geometric solution

Corresponding Author: Professor Myles Lewis

**This file contains all reviewer reports in order by version, followed by all author rebuttals in order by version.**

Version 0:

Reviewer comments:

Reviewer #1

(Remarks to the Author)
The authors present cellGeometry, an R package for reference-based deconvolution of bulk RNA-Seq using single-cell atlases. The method employs "non-negative geometric deconvolution" (NGD), which performs vector projection of bulk samples against cell-type signature vectors, followed by spillover compensation with per-cell-type damping to ensure non-negative outputs. The manuscript emphasizes computational speed gains through matrix operations, benchmarks against MuSiC/DWLS/LinDeconSeq on pseudo-bulk simulations, and validates on whole blood and RA synovium cohorts. I believe that the method is a practical engineering contribution with clear speed advantages for atlas-scale references, but the core algorithmic novelty is overstated. NGD is essentially weighted least squares with a heuristic non-negativity constraint, and the manuscript does not convincingly establish that it represents a fundamentally new paradigm rather than an incremental variant of constrained regression with post-hoc regularization.

Major Concerns

1. The authors acknowledge in the Methods (lines 926-1018) that NGD has a "partial relationship to ordinary least squares" and is "equivalent to a weighted least squares solution." The core operations of vector projection via dot product, sphere scaling for equal gene weighting, spillover matrix as Gram matrix and compensation as matrix inversion, are all standard linear algebra from regression theory. The manuscript's geometric language (vectors, angles, projection, orthants) obscures rather than clarifies what is fundamentally $\beta = (X'X)^{-1}X'y$ with non-negativity post-processing.
The only distinctive component is variable compensation (tuning w_j per cell type to prevent negative outputs). However, this is presented as a heuristic optimization (golden section search to find w where $\min(\text{output})^2=0$) rather than derived from a principled objective. The manuscript states it is "related to, but distinct from, ridge regression" without formalizing exactly what NGD optimizes.

2. Given that NGD is essentially constrained least squares, the most relevant baselines are non-negative least squares (NNLS), NNLS with Tikhonov/ridge regularization, and other methods that explicitly enforce $\beta \geq 0$ during optimization rather than post-hoc. The manuscript only compares to MuSiC, DWLS, and LinDeconSeq (all W-CLS variants), plus NMF. NNLS is well-established, has fast implementations (e.g., nnls, glmnet with lower.limits=0), and directly solves the non-negative regression problem that NGD approximates with variable compensation. Without this comparison, it is unclear whether NGD's variable compensation offers accuracy improvements over simply using NNLS from the start.

3. All accuracy simulations use pseudo-bulk generated by Dirichlet sampling from the same scRNA-Seq dataset used to construct signatures (with arithmetic mean for simulations). This is an idealized setting where the reference perfectly matches the target. The noise experiments add perturbations but do not simulate domain shift where bulk comes from different chemistry, different cohort, or has cell types/states not in the reference.
The manuscript emphasizes robustness to "chemistry differences" but supports this primarily with noise transformations (log/sqrt/shift noise) rather than true cross-platform experiments.

4. The manuscript describes iterative removal of genes with extreme Studentized residuals, presented as a diagnostic/correction mechanism. However, removing genes will always reduce residuals and SE. This does not guarantee improved deconvolution accuracy. In fact, removing true biology (e.g., interferon-response genes upregulated in disease) could bias estimates toward the reference state.
The blood example shows removal of granulocyte/RBC markers and interferon genes (interpreted as disease-relevant signal).

But no quantitative validation demonstrates that iterative removal improves correlation with ground truth versus single-pass deconvolution.

5. The novel SE estimator based on cross-sample residual variance is interesting, but the manuscript only validates it via correlation with true error. Correlation indicates the SE tracks relative error magnitude but does not establish that the SE has correct absolute scale or frequentist calibration.

6. DWLS failed on 80.3% of R4RA synovium samples, reportedly due to quadratic programming breakdown. While this is a real usability limitation, the manuscript uses this to imply DWLS is an inferior method rather than simply unsuitable for this particular reference/bulk combination. DWLS performed comparably to cellGeometry in simulations.

7. The manuscript emphasizes ability to deconvolute 18-461 cell subclasses, but validation against external measurements is limited to:
- Coulter counter: only neutrophils vs. lymphocytes (2 broad categories)
- Histology: CD3/CD20/CD68/CD138 (4 markers, semi-quantitative scoring)

Neither validates the fine-grained T/B cell subsets, monocyte subtypes, or fibroblast subpopulations that constitute most of cellGeometry's output. The correlation with Coulter/histology demonstrates consistency at the coarse level but cannot confirm accuracy for the detailed subclass estimates that differentiate this method.

8. The Methods explicitly state the algorithm "assumes that each cell subtype is mutually distinct since it treats their dimensions as orthogonal". However, many biologically relevant cell states exist on continuums (Monocyte → macrophage differentiation; Naive → memory → effector T cell trajectories; Activation states within cell types). When applied to references with granular clustering (e.g., Tabula Sapiens with 461 subclasses), this assumption is systematically violated. The paper shows accuracy degrades with similar cell types but frames this as a limitation of the reference rather than the method. This is a fundamental constraint that affects interpretation.

9. All benchmark comparisons are presented as boxplots without formal statistical testing. Given that differences in RMSE/$R^2$ between methods are sometimes modest (e.g., cellGeometry vs. DWLS in simulations), it is unclear which differences are statistically significant vs. sampling variability.

10. cellGeometry has numerous tunable parameters (noise filter ratio, expression filter, number of genes per subclass, compensation settings) that were presumably optimized. The baseline methods were run with "default settings". This asymmetry could favor cellGeometry.

Minor Concerns

1. The abstract claims cellGeometry is "more accurate than existing methods" but benchmarks only include three methods from one comparative review. This is an overclaim given the >50 deconvolution methods in the literature.

2. Runtime comparisons lack hardware normalization. MuSiC cannot parallelize while cellGeometry uses 8 cores. Report single-core times for fair comparison, or normalize to core-hours.

3. Gene selection by vector angle is intuitive but the number of genes selected per cell type, whether sets are disjoint/overlapping, and sensitivity to this parameter are not clearly reported.

4. The "noise filter" (reducing low-level expression to zero) is described briefly but its effect is not ablated. What ratio threshold is used by default?

5. Merging signatures via QQ mapping assumes distributions are similar. Provide sanity checks that merged signatures preserve expected marker relationships; potential distortions are not discussed.

6. "Cell counts" vs "proportions" terminology is inconsistent. Most methods output proportions; cellGeometry outputs counts. Clarify the output scale and provide guidance on normalization for comparison.

7. The cosine similarity heatmap is mentioned but the actual heatmap is tiny/unreadable in the figure. Critical for interpreting deconvolution limits.

8. Histology validation shows correlations but acknowledges thin-section sampling limitations. Consider emphasizing this caveat more prominently when interpreting "accuracy."

9. The claim that cellGeometry "redefines pathotypes" is potentially overstated. It refines pathotype classification using deconvolution-derived thresholds, but histology remains an independent modality.

10. The Methods state $\log_2$+1 transformation for signature generation but arithmetic mean of raw counts for deconvolution of simulated data. This inconsistency in scale should be more clearly justified.

(Remarks on code availability)
Yes. The package is well-documented and fully functional. Standard users can install and run the software following the provided vignettes. Full reproducibility of paper benchmarks requires the authors to make the benchmark repository public (https://github.com/EMR-bioinformatics/cellGeometry_manuscript/ is unavailable).

Reviewer #2

(Remarks to the Author)
1. Summary
This manuscript presents cellGeometry, a bulk RNA-seq deconvolution framework built from single-cell–derived signatures, framed through an explicit spillover/compensation view: a spillover matrix S captures cross-talk among reference cell types and the method uses compensation (conceptually S^{-1}) to correct spillover. A central technical contribution is variable compensation with non-negativity control, introducing a per-cell-type damping weight vector w in [0,1]^n to avoid negative abundance estimates while retaining much of the spillover correction. The paper also includes (i) a marker specificity/visualization scheme using unit-hypersphere scaling and vector angles, (ii) a residual-based uncertainty proxy ("var.e") and related SE options, and (iii) a residual/outlier workflow that can highlight reference incompleteness and composition-adjusted biological programs (e.g., IFN-related residuals). The authors demonstrate utility in RA blood and RA synovium, including histology-grounded validation and a bulk-based redefinition of synovial pathotypes.

2. Strength
- Conceptual clarity and interpretability: The spillover/compensation framing makes the failure mode of closely related cell types concrete, and the non-negativity issue is handled via an explicit, inspectable mechanism (w-damped compensation).
- Operational robustness in difficult regimes: The manuscript documents settings where QP-based approaches can fail to return solutions under certain noise perturbations, while cellGeometry remains stable.
- Scalability is a genuine differentiator: The reported runtime improvements at thousands of samples and atlas-scale settings are substantial and align with the method's matrix-operations design.
- Strong disease-relevant validation: In RA synovium, deconvolved abundances correlate with orthogonal histology markers and show improved biological plausibility relative to several baselines.
- Diagnostics beyond point estimates: The residual/outlier pipeline is practically useful: it flags missing cell types (e.g., granulocyte/RBC marker residuals when absent from reference) and can surface disease-state residual programs (e.g., IFN response) after accounting for composition.
- Uncertainty is addressed explicitly: The "var.e" approach is well-motivated and shown to correlate well with error in simulations, and the link to cell-type similarity is sensible.

3. Major Comments
3.1 Formalize the w-tuned variable compensation as an estimator, and add a stability diagnostic (conditioning): The non-negativity mechanism via w is the most distinctive algorithmic element. I recommend strengthening its statistical interpretability by (i) providing an explicit objective-function view (even approximate), and (ii) quantifying the bias/variance tradeoff relative to NNLS/QP baselines in high-collinearity regimes. In addition, because spillover correction is fundamentally limited by collinearity, I strongly suggest reporting a condition-number (or spectral) analysis of the spillover/Gram matrix (e.g., kappa(S)) across benchmarks and plotting it against deconvolution error. This would make the "not-resolvable subtype" regime explicit and give users a practical diagnostic for when subtype-level claims are not identifiable.

3.2 Elevate domain shift to a primary benchmark: Noise-robustness and operational stability are important, and the RA synovium validation is compelling. However, the main failure mode in real deployments is reference misspecification/cross-study shift (chemistry, dissociation, donor effects, disease vs healthy). A flagship "reference A -> bulk/pseudobulk B" evaluation (different cohort/lab/chemistry) would substantially strengthen the generality claim.

3.3 Validation metrics: add calibration-sensitive reporting (beyond correlation) and calibrate uncertainty. Some validation relies on correlation-style metrics or correlation-driven interpretation. Because Pearson correlation can mask systematic scale/shift bias in absolute abundance, I recommend adding Lin's Concordance Correlation Coefficient (CCC) and identity-line calibration (slope/intercept or Bland–Altman) on key orthogonal validations. Separately, while var.e correlates with error in simulation, I would add coverage-style calibration of intervals (nominal 90/95%), especially stratified by cell-type similarity, to make uncertainty actionable.

3.4 Make the residual "state after composition" layer a first-class analysis product.
The residual/outlier workflow is one of the most practically valuable contributions. I suggest formalizing this as a deliberate second layer ("composition-adjusted residual programs"), with explicit guidance on distinguishing technical mismatch (missing reference components) vs biological state shifts, and at least one replicated cohort example to demonstrate stability.

3.5 Pathotype redefinition: justify thresholds and assess stability.
The bulk-based RA synovium pathotype redefinition is appealing, but hard thresholds (e.g., B cells > 30; macrophages > 300) raise concerns about protocol dependence and biopsy cellularity. A sensitivity analysis over thresholds (and/or normalization considerations) would strengthen the translational story.

4. Minor Comments
- Provide a compact algorithm box that standardizes notation across Y, G, H, S, compensation, and w, and clarifies what is computed in count vs log space (since this impacts additivity and interpretation).
- The vector-angle marker ranking is clear and useful, but should be explicitly positioned as cell-type specificity rather than state-program discovery (to avoid over-interpretation in continuous-state settings).
- Provide recommended defaults and stopping criteria for iterative extreme-residual removal, and clarify how it interacts with compensation tuning.

- Make benchmarking conditions maximally explicit (hardware, parallelization, preprocessing parity) given the very large runtime differences reported.

5. Overall
This is a strong, well-motivated, and practically impactful methods manuscript. The combination of (i) explicit spillover/compensation geometry, (ii) a pragmatic non-negativity mechanism, (iii) strong RA synovium validation with orthogonal support, (iv) a genuinely useful residual diagnostic layer, and (v) standout scalability makes it compelling as more than an incremental engineering exercise. The main upgrades that would most materially elevate the manuscript's impact and perceived rigor are: (1) an objective-function and bias/stability characterization of w-tuned compensation plus a conditioning diagnostic, (2) a headline domain-shift benchmark, and (3) calibration-sensitive validation metrics (CCC + identity calibration) alongside calibrated uncertainty. With these additions, I would be comfortable advocating this manuscript for Nature Comms.

(Remarks on code availability)
- Code availability appears strong and reproducible, with extensive documentation and vignettes
- I was able install the package out of the box and was able to explore a few vignettes without any issue

Reviewer #3

(Remarks to the Author)
The authors present a deconvolution method called cellGeometry that uses matrix operations for a faster deconvolution. The authors test the method's performance in simulated bulks and real bulks, and compare results to other well-performing methods, across different tissue types. CellGeometry consistently is shown to be faster and outperform other methods. The method's implementation is modular and is offered as an R package for easy use. The authors are very thorough in their benchmarking and offer all information in figures.

Comments:

- In the section "Ability to merge single-cell reference signatures" and others, the authors describe cellGeometry as having the "unique ability" to merge gene signatures from different scRNA-seq datasets. Then the authors say MuSiC does not have the feature of merging single cell clusters, and omit comparison analysis because of this. Can you elaborate on what this means? MuSiC is specifically for multiple subjects/datasets, and simply changing cell type names could help create the desired gene signature, and adding batch or patient ID will likely increase accuracy if not currently implemented. I would suggest the authors address this in the revised version of the manuscript.

- I suggest the authors corroborate that each method is used as intended. For example, like mentioned above, MuSiC is meant to have multiple subjects, so patient/batch information should be passed to the model. Furthermore, the authors later write some cells were used with only 4 cells in the single-cell reference. This is contrary to the specifications of most methods that exclude analyses when there are less than 10 cell types. For example, MuSiC's reliable estimation depends on sufficient sampling to estimate cross-subject means and variances. The DWLS failures are due to the method not being used as expected mathematically (too few cells per example), not a method failure but a user error. If cellGeometry is able to deconvolve accurately with such a low number of cells per cell type, then that is a big advantage of the method. However, the other methods should be used as intended for fair comaprison. I would suggest rerunning the analyses with this information, or address this by adding an analysis testing number of cells in reference vs. performance. Cell types can be merged for usable cell type numbers if needed. Outside of showcasing the ability to deconvolve very granular cell types, I don't see a need to use cell types with less than 25-75 cell examples across multiple datasets, especially since at that level of granularity most real bulk samples will not have all states of a particular cell type, and reporting all of them in real bulk is concerning (detecting "all 18 subclasses in the synovium" could likely be cellGeometry error rather than true presence of cells).

- Related to the above comment, there is research showing that increasing the number of cells per cell type, or number of cells in the reference, does not increase deconvolution accuracy in some methods (accuracy plateaus past a certain point). The authors sometimes omit analyses based on R running out of memory, etc. However, it could be that including less cells per cell type (controlled in the proper manner) could 1. Make those analyses possible in R, particularly those that failed DWLS, and 2. Would increase accuracy of all methods. I would suggest the authors address this in the revised version of the manuscript.

- The authors have no differentiation of single cell and single nucleus RNA-seq datasets within the Human Brain Atlas (could be others). It is commonly reported that neurons and other cell populations are not able to be observed in single nucleus datasets, so I assume the datasets used contain both, combined Single cell references and single nucleus references can impact deconvolution in most methods, and I would suggest the authors consider this.

- The use of results to adjust gene signatures and improve deconvolution is very interesting. Out of curiosity, have the authors applied this to other deconvolution methods as well? i.e., would DWLS also improve accuracy with multiple passes after gene filtering? Could other methods also benefit?

- There are many supplementary figures showing real vs. predicted proportions, and some of these take over multiple pages. The manuscript would be more digestible if the authors made the figures only be one page at the most, and merged some of the panels to minimize the number of supplemental figures. You can try multiple cell types in one plot, with different shapes or colors. Also, it is concerning that some of the values in the supplementary figures showing predicted vs. "real" cell type proportions go past the 1.2 tick mark. The assumption is proportions sum to 1, so cell types can not have a proportion higher than 1, specially in mixture. I would kindly ask the authors to explain why values go past 1 in real proportions.

- It seems from Fig. 6B that MuSic best aligns with the cell populations expected in bulk RNa-seq samples, even if cellGeometry detects more cell types (which is not what one would expect in bulk RNa-seq). The manuscript would be strengthened if the authors addressed this.

- In Supp. Fig. 18b, the Gaussian noise increase causes no change in Rsq. This is very contrary to what is usually observed in models like DWLS, MuSiC. Do the authors have an explanation for seeing no change in performance? And could the authors also explain what are the values in the x axes of these figures (Gaussian noise from 0-500)? It would be worth shortening some of the figure legends by removing redundant information to help make the axes/figures more interpretable, keeping only the needed information. If something is reported as results, there is no need to report again in figure legends.

- I recommend the authors be more cautious about using the words “novel” or “unique” throughout the manuscript. For example, using word novel when describing the variable compensation nnmf and also describing var.e SE measure.

- The authors show in Fig. 5 the decreasing residuals with more passes. I would kindly suggest adding a label to what the red values are vs. black values in the Fig. 5a plots. And can you make it clear how exactly the outlier genes are classified as outlier? a threshold?

- In Fig. 6c, the authors show the correlation of marker expression vs. calculated cell abundances/proportions for 4 cell groups. Is there a reason this analysis was done with these 4 groups rather than the same 4 groups explored in Fig. 6b? Both panels would be strengthened by using the same 4 groups. Also, the correlation coefficients are very close in some cases, making the results comparable, and this is not addressed in results (only cellGeometry outperformance is mentioned).

- After reviewing the manuscript, the question remains of whether the very granular identified cell populations (multiple subsets of T cells, etc.) truly represent real cells within real bulk. The manuscript would be strengthened by showing cellGeometry and other methods with negative controls: “if a pseudo-bulk does NOT include X granular cell type/state, is the deconvolution method still predicting it?”.

Minor:

- Consistency in paragraph indentation, italics genes, and acronym introductions.
- Sentences in formal writing typically don’t start with numbers or lower case letters.
- References for deconvolution methods in introduction. For example, MuSiC is not cited.
- Make sure all labels in all figures are readable. For example, Supplementary Fig 2 axes labels hard to read.
- Figure 6A would greatly benefit if the pathotype were ordered in each panel, in same order across panels, to be able to compare groups visually, and Figure 6B needs y axes labels (Cell freq either percentage, cell number, etc for each method).
- FACs not defined when first introduced, and a reference is needed to validate that sentence.
- Please make sure every statement made throughout manuscript, introduction and discussion particularly, is backed-up with at least one reference (for example: in discussion the authors say deconv. methods “fail outright”). If the authors are referribng to DWLS’s failure in the current study, that was a cell number issue from the authors and not a DWLS failure.
- NGD acronym is defined multiple times.

(Remarks on code availability)
The above link is to the R package page, not the code. The manuscript has the link: https://github.com/myles-lewis/cellGeometry , which does contain the code for the package with a README with instructions on how to use it. however, this repository doesn't have the instructions to replicate the study results, even if the method's code is there.

Version 1:

Reviewer comments:

Reviewer #1

(Remarks to the Author)
I thank the authors for their substantive revision. I am satisfied with the responses to the majority of my points. Two issues remain where I believe the wording needs adjustment in the abstract:

1. Framing of NGD as a novel regularisation - The authors now show explicitly that NGD reduces to a constrained generalized Tikhonov problem with a heuristic, non-negativity-driven choice of the regularisation weight vector w rather than a CV-tuned scalar λ. This is a legitimate and useful variant, and I appreciate the added derivation. However, the abstract, introduction, and discussion still describe NGD as "a novel form of non-negative matrix regularisation" and as a "new paradigm". Given the derivation now in the Methods, I would ask the authors to align the framing with the math: position NGD as a constrained generalized Tikhonov method with a novel heuristic for the regularisation weights (driven by non-negativity of cell counts rather than cross-validation), rather than as a distinct paradigm. The scientific contribution is not diminished by this rewording, if anything, it is clarified.

2. Generalisability claims given the absence of cross-platform validation - I accept the authors' argument that truly matched cross-platform (same-cells). However, the abstract still states cellGeometry "is robust to biases introduced by different sequencing chemistries", and the discussion generalises from pseudo-bulk simulations plus one synovium real-data

validation. A reference-A to bulk-B design across independent cohorts/chemistries is not impossible and remains the cleanest test of the generality claim. At minimum, I would ask the authors to soften the chemistry-robustness language in the abstract and discussion to reflect that robustness has been demonstrated primarily through simulated noise models (log noise, shift noise) rather than empirical cross-platform data, and to explicitly acknowledge this as a limitation.

(Remarks on code availability)
I didn't test the code.

Reviewer #2

(Remarks to the Author)
Breakdown of my previous concerns:

Major comments:

3.1 Estimator / conditioning / NNLS comparison: largely addressed.
3.2 Domain shift as a primary benchmark: only partially addressed, but the response is reasonable.
3.3 Calibration-sensitive validation: mostly addressed, but incompletely. My request for interval calibration, especially nominal 90/95% coverage and stratification by cell-type similarity, was not addressed.
3.4 Residual layer as a first-class product: partially addressed. The residual workflow is now clearer and better supported, although it is not fully developed into the broader framework I had suggested.
3.5 Pathotype thresholds / stability: adequately addressed.

Minor comments: mostly addressed.

Overall: The revision has addressed most of my major concerns and has materially improved the manuscript. The authors have strengthened the statistical formulation of the method, added additional baseline and calibration analyses, and made the pathotype-thresholding procedure more data driven and defensible. One major concern remains only partially addressed, but there is no longer any issue that precludes publication, and I would support acceptance.

(Remarks on code availability)
I checked the public CRAN release of the package and verified from the package documentation that the main code-facing additions described in the revision are now present, including expanded deconvolution options, residual diagnostics, SE extraction, conditioning diagnostics, and signature-merging utilities. I did not independently rerun the full manuscript benchmarking pipeline, so this should be taken as confirmation of public implementation and documentation rather than a full reproducibility audit. The code availability is therefore substantially improved and, in my view, adequate for publication.

Reviewer #3

(Remarks to the Author)
1.We are specifically referring to merging of 2 distinct scRNA-Seq datasets. While this is possible manually, it requires aligning the rows of genes and binding columns of the 2 raw SC count matrices together, then concatenating the SC metadata, and adding a column for batch. However, that compute process is not trivial when the count matrix and metadata run into 30 GB file sizes, where the finalised merged objects need to be saved to disc. MuSiC has no way to specify multiple scRNA-Seq dataset objects as input. In our simulation of the brain dataset the simulated pseudo-bulk is generated by sampling from both the neuronal and non-neuronal SC datasets and then summing the resulting bulk gene expression. We show that analysis of the neuronal and non-neuronal datasets by MuSiC is i) extremely time consuming and ii) inaccurate. cellGeometry takes a different view. It merges only the signature matrices, not the raw single-cell count matrices. This is considerably more convenient for users and avoids manipulations of massive on-disc matrices. If the signature matrix may have a slightly different scale (this varies depending on number of cycles of PCR etc during library prep), the distributions of the signature matrices are aligned using QQ mapping before merging. With the brain atlas datasets, QQ mapping is not required as the 2 SC datasets were generated by the same lab using the same scRNA-Seq methodology. In this situation cellGeometry is able to deconvolute simulated bulk brain RNA-Seq containing both neuronal and non-neuronal cells without degradation in accuracy.

The authors' response does not address my concern. A valid method comparison requires each tool to be evaluated within its intended use case. MuSiC was not designed for atlas-scale, multi-dataset deconvolution, and benchmarking it in that setting is circular: the experimental framework was designed around cellGeometry's strengths. If dataset size is the barrier to running MuSiC appropriately, the solution is to downscale the dataset to a size where MuSiC can operate as intended, then perform the comparison with cellGeometry. If cellGeometry still outperforms in accuracy under those conditions, that is a legitimate result worth reporting. The authors should either perform the comparison on an appropriately scoped dataset, or remove the superiority claim and reframe cellGeometry's atlas-scale capability as a novel extension rather than a benchmarked advantage.

2. cellGeometry removes cell subclasses with <10 cells in the scRNA-Seq by default since we believe there is insufficient information if fewer than 10 cells are present. This cutoff can be controlled by users through a parameter setting. The authors of DWLS state that one of the rationales of DWLS was to overcome rare cell population deconvolution, but they do not specify how rare. Both MuSiC, DWLS and LinDeconSeq give no specific instructions for removing cell subclasses below a particular

threshold. MuSiC2 was designed with 2 bulk tissue datasets in mind (control vs disease) to be able to handle the situation where some cell subclasses might be rare and only present in one bulk dataset. However, we could not find a way to use this method as we did not have matching control vs disease bulk datasets and had no easy way to simulate this. MuSiC2 removes cell subclasses where the mean proportion of cells across all samples in the bulk data is < 10% of the cells as the default (prop_r = 0.1). This means it is mainly designed only to detect much more common cells and will remove rare cells in bulk by default. MuSiC1 & 2 do not specify a cutoff for the number of cells in the scRNA-Seq dataset. We re-ran MuSiC, LinDeconSeq and DWLS for the deconvolution of real-world whole blood bulk RNA-seq whilst removing single-cell clusters with <10 cells in the reference. This hardly altered their deconvolution results (Table R1 below) and the correlation between predicted lymphocyte percentages and lymphocyte Coulter Counter measurements did not improve (Fig. R7 below). In the AMP synovium dataset, the minimum number of cells per subclass was 48. We considered all of these subclasses to be important and functional, as described in the AMP paper (Zhang F 2019, Nature immunology, PMID: 31061532). As we believe from earlier histological studies that we are likely to find all 14 of these cell types within inflamed rheumatoid arthritis synovial tissue (albeit not in all patients all of the time), we did not remove any of these subclasses. We provide the bulk synovium RNA-Seq dataset for users to test out DWLS for themselves.

The authors' response does not address my concern, which is still related to the one above. Comparing methods in a way they are not intended to be used, then claiming cellGeometry superiority in accuracy etc. If the authors want to keep their current methodology in benchmarking (i.e., atlas-level scales of data etc.), please choose methods that are intended to be used at this level of data. Bisque and InstaPrism are 2 options that can serve in this framework when comparing performance at atlas-level scale.

3-To overcome the matrix limit of 231 elements, we restricted the maximum number of cells to a fixed amount and sampled for the cell subclasses randomly to stay within the 231 limit. This reduced each of the 1-3 million cell scRNA-Seq datasets to around 20-30,000 cells. The subsetted Human Brain Cell Atlas and Tabula Sapiens datasets were used to assess deconvolution by DWLS and LinDeconSeq. In spite of subsetting the cells, LinDeconSeq was unable to deconvolute both Human Brain Cell Atlas (neuron) and Tabula Sapiens. It failed at the signature generation step. Full details and a reprex script have been provided on the Github repo:
https://github.com/EMRbioinformatics/cellGeometry_manuscript/tree/main/02_Stimulated_benchmarking/No_noise/LinDeconSeq_reprex Even with the scRNA-Seq subset to 20-30,000 cells, DWLS took 54 hours to generate the gene signature for Tabula Sapiens. Although DWLS had high accuracy for deconvoluting Tabula Sapiens, DWLS could only consider neuron and non-neuronal cells separately and so did not perform as well (main Fig. 1c [revised]). Human Brain Cell Atlas non-neuronal simulated data was deconvoluted using DWLS and LinDeconSeq where both did not perform well and were significantly worse than cellGeometry (Supplementary Figure 17).

I understand that subsetting to 20-30,000 also caused the comparison methods to break down mathematically. This is expected; a scRNA-seq reference in practice would very rarely have more than 2,000 per cell type. If the programming language is the issue, most methods have a Python implementation, or other methods can be tested. DWLS is notoriously noted as a slow method not meant for large datasets in benchmarking papers (https://www.nature.com/articles/s41467-020-19015-1). To highlight cellGeometry's ability to deconvolve 1. Fast and 2. Using atlas level dataset, it would be much better to compare to methods that are meant for this, like InstaPrism, Bisque. This is related to my comments above; appropriate comparison would strengthen the manuscript.

4. The Human Brain Cell Atlas is single-nucleus RNA-seq, whereas the other datasets (AMP rheumatoid arthritis synovium, Cell Typist and Tabula Sapiens) are single cell. We have clarified this in the text.

I believe this to be a larger concern than stated. There are methods specifically designed to use single-nucleus RNA-seq references as reference. Is this also the intention with cellGeometry? Increasing research has shown single-cell and single-nucleus are not interchangeable as references. The manuscript would benefit from explicitly experimentally showing single-nucleus is an effective reference for this cellGeomtry, or comparing the single-nucleus performance to methods that also use single-nucleus rather than methods that use single-cell.

5. Using the genes that were filtered out by cellGeometry for the deconvolution of whole blood RNA-seq from the PEAC clinical study using the Cell Typist blood dataset (as described in main Fig. 5a-c), we have removed them from the DWLS signature and from the Cell Typist blood reference for MuSiC. MuSiC is a one-step deconvolution process so the signature cannot be directly intercepted and adjusted accordingly. It was noted that LinDeconSeq gene signature already did not contain the genes filtered out by cellGeometry. 16 With these adjustments, whole blood RNA-seq deconvolution was rerun for MuSiC and DWLS (Fig. R8 ab below) where we generate same comparisons as main Fig. 5e & g (for ease of comparison Fig. R8 c-d below). The correlation of lymphocyte percentages with Coulter counter measurements improved slightly, but was still not as good as cellGeometry (r = 0.53, p = 3.3e-05). We have noted in the manuscript text that gene filtering of the gene signatures in other deconvolution methods was performed, but only partially improved accuracy. Both MuSiC and DWLS still overestimated monocytes and underestimated T cells.

The authors have addressed my comment.

6. These were cell subclass percentages rather than fractional proportions which add up to 1. The legend has been amended to clarify. The correlation plots for Tabula Sapiens and the Human Brain Cell Atlas simulated data have been consolidated into single plots for each deconvolution method (Supplementary Fig. 9-11).

The authors have addressed my comment.

7. This is addressed in Fig. 6c where the MuSiC estimated T cells do not correlate with CD3 histology.

Thank you for clarifying. However, those values appear to be very close. Is there a statistically significant difference?

8. There are 2 main reasons:
i) Since the RNA data is on count scale, Gaussian noise barely touches genes with high expression e.g. whose counts run into 10^5 or more. As these genes are untouched and often more highly leveraged in deconvolution methods, highly expressed genes provide full information for the algorithm.
ii) Gaussian noise does not change Rsq unless the noise is very, very large because the
noise is unbiased. So on a population level most methods use many genes (equivalent to large n) and if each gene a b MuSiC DWLS MuSiC DWLS c d Rerun with gene filtering Original 17 is affected equally by noise, then if the deconvolution method is BLUE and so based on averaging inference from many genes it is not affected much by Gaussian noise. Noise methods with intrinsic bias will more heavily affect these systems. Non-Gaussian noise (log, sqrt) will be upwardly biased once converted back to count scale. Because shift_noise selects genes at random, this causes bias in whole genes. If multiple genes which are cell-specific are shifted this will alter the deconvolution in a biased, nonsymmetric way, so methods are more sensitive to this.
I want to tank the authors for their explanation, but have concerns about all arguments presented.
i) This is inconsistent with how these methods operate. MuSiC explicitly downweights broadly highly expressed genes, and DWLS is built around marker gene selection. The genes driving deconvolution are cell-type-specific markers, not globally highly expressed genes, so this argument should be revised or removed.
Regarding point (ii) and the figure more broadly: While the unbiasedness argument is statistically sound, an equally plausible explanation for the flat $R^2$ is simply that the Gaussian noise levels tested (SD=100–500) are trivially small relative to the dynamic range of the data, which from Supp. Fig. 18a clearly spans up to $10^8$ counts. The two explanations cannot be distinguished from the data as presented. The authors should test substantially larger Gaussian noise levels to determine whether the flat $R^2$ reflects true robustness or merely insufficient noise magnitude. As currently presented, this analysis does not convincingly support either conclusion.

(Remarks on code availability)
The code is present in the repository, but no instructions on replicating the experiments are included.

Version 2:

Reviewer comments:

Reviewer #2

(Remarks to the Author)
Assessment of whether Reviewer #3's remaining concerns have been addressed:

I reviewed the revised manuscript, rebuttal, and supplementary material with the specific goal of assessing whether the remaining concerns raised by Reviewer #3 have been adequately addressed. Overall, I think the authors have now addressed the main substance of Reviewer #3's concerns. The original concern about fairness in the atlas-scale benchmarking was valid, but the revised manuscript now includes the key missing controls: downsampled MuSiC, Bisque, and InstaPrism. These additions make the benchmarking much more defensible. I do not see a remaining Reviewer #3 issue that should block publication. I would still ask for a few small wording and correctness edits.

1. Atlas-scale benchmarking and MuSiC comparison:

Reviewer #3's main concern was that the original comparison was not fully fair: cellGeometry was being tested in a setting that used its strengths, especially very large references and merging separate references, while MuSiC was not designed for that exact use case.

The authors have now addressed this in a reasonable way. They reran MuSiC on downsampled Tabula Sapiens and Human Brain Cell Atlas references, using the same broad downsampling strategy used for other methods. This was the correct control to add. The downsampled MuSiC results do not close the accuracy gap, so the original conclusion is now better supported.

The authors also added Bisque and InstaPrism, which directly addresses Reviewer #3's request to include methods more appropriate for atlas-scale or large-reference settings. These comparisons are now included in the supplementary figures and Supplementary Table 1. This substantially improves the fairness of the benchmark.

My only remaining issue is the wording around the Human Brain Cell Atlas result. In that analysis, cellGeometry is not only being compared as an estimator; it is also being tested for its ability to merge neuron and non-neuronal reference signatures. That is a real and useful feature. But it is not a perfectly apples-to-apples estimator comparison, because the other methods are not set up to merge signatures in the same way. I would ask the authors to frame this result more explicitly as a demonstration of cellGeometry's merged-reference capability, rather than only as a pure accuracy benchmark.

Note: this is a wording issue, not a request for new analysis.

2. Comparator methods for atlas-level data: This concern is addressed.

Reviewer #3 specifically suggested Bisque and InstaPrism. The authors added both. The supplementary table and plots show that these methods did not outperform cellGeometry on the atlas-scale benchmarks. This is the right response.

One small wording point: the phrase “purportedly designed for large-scale sc/snRNA-Seq studies” sounds a little dismissive. I would suggest replacing it with something more neutral, such as “methods applied to large-scale sc/snRNA-Seq studies” or “methods intended for large-scale reference-based deconvolution.” The authors do not need to weaken the result, but the wording should stay neutral.

3. Downsampling and intended use of methods: This is mostly addressed.

The authors now explain how large references were downsampled for methods that could not handle the full matrix size. They also tested downsampling for MuSiC. That addresses the main fairness issue.

I would still keep two points separate in the manuscript: 1st, cellGeometry scales better computationally; 2nd, cellGeometry is more accurate in these benchmarks. These are related, but they are not identical claims. The revised manuscript mostly makes this clear, but the brain atlas section still risks mixing “feature advantage” with “estimator accuracy.” This can be fixed by wording.

4. Single-nucleus RNA-seq reference issue: This is the only Reviewer #3 concern that is not fully experimentally resolved. But I think the authors’ response is acceptable.

Reviewer #3 correctly pointed out that the Human Brain Cell Atlas is single-nucleus RNA-seq, and that scRNA-seq and snRNA-seq references are not interchangeable. The authors now state in the main text that the benchmark includes three scRNA-seq datasets and one snRNA-seq dataset. They also added limitation language explaining that a proper test would require matched scRNA-seq, snRNA-seq, and bulk RNA-seq from the same samples.

I agree that such a matched experiment is beyond the scope of this revision. I would not block the paper on this point. However, the manuscript needs to be careful and consistent. The current evidence supports that cellGeometry can be applied to the Human Brain Cell Atlas snRNA-seq reference in this simulation setup. It does not prove that snRNA-seq references are generally validated for real bulk deconvolution across tissues.

I would ask for small wording corrections wherever the Human Brain Cell Atlas is included. Some text still uses “scRNA-seq datasets” generically when the Human Brain Cell Atlas is part of the comparison. This should be changed to “sc/snRNA-seq datasets” or “single-cell/single-nucleus reference datasets.” This applies especially to the abstract, figure legends, and Supplementary Table 1 caption. This is a correctness issue.

5. Gene filtering / residual gene removal: This concern is addressed.

Reviewer #3 asked whether cellGeometry’s advantage could come from its residual gene filtering rather than the deconvolution method itself. The authors tested removing the same outlier genes from the inputs/signatures used by other methods and found only small improvements. This is an adequate response.

6. Percentages versus proportions: This is addressed.

The revised legends and supplementary plots now clarify that the metrics are based on predicted versus true cell percentages/subclass percentages. I have no further concern here.

7. Statistical comparison of histology correlations: This is mostly addressed.

Reviewer #3 asked whether the apparent difference between cellGeometry and MuSiC in the T-cell/CD3 histology comparison was statistically significant. The authors report in the rebuttal that the difference is significant by Fisher r-to-z test.

That answers the question. However, I think the Fisher r-to-z result should be added to the manuscript or supplement, not only left in the rebuttal. This is a small edit, but it would make the statistical clarification visible to readers.

8. Gaussian noise analysis: This concern is addressed.

Reviewer #3’s objection was logical: the original Gaussian noise levels may have been too small relative to the dynamic range of the count data, so the flat performance curve might not mean true robustness. The authors responded by testing substantially larger Gaussian noise levels and updating the main and supplementary figures. The revised plots now show performance changes as Gaussian noise increases.

That resolves the concern.

I would only avoid overexplaining why MuSiC or LinDeconSeq curves are flatter unless that mechanism is directly tested. The revised manuscript does not need much speculation here. It is enough to say that larger Gaussian noise levels were tested and that the main conclusions are unchanged.

9. Code availability and reproducibility instructions: This is adequately addressed for publication.

The authors now point users to CRAN vignettes and provide benchmarking/figure-generation scripts. I did not rerun the full benchmarking pipeline, so this is not a full reproducibility audit. But the code/instruction issue raised by Reviewer #3 has been improved enough.

Overall assessment

I think Reviewer #3's main concerns have been substantively addressed. The most important fixes were the downsampled MuSiC analysis, the addition of Bisque and InstaPrism, the expanded Gaussian noise benchmark, the gene-filtering control for other methods, and clearer code/vignette information.

The only point that remains partially addressed is the snRNA-seq issue. The authors did not add matched scRNA/snRNA/bulk validation, but they now acknowledge this limitation. I think that is acceptable for this manuscript, as long as the claims are cautious and the terminology is corrected.

I would support acceptance after minor wording/correctness edits:

1. Frame the Human Brain Cell Atlas result as a merged-reference capability result, not a purely apples-to-apples estimator comparison.
2. Use "sc/snRNA-seq" or "single-cell/single-nucleus reference datasets" consistently wherever the Human Brain Cell Atlas is included, including the abstract, figure legends, and supplementary table captions.
3. Add the Fisher r-to-z comparison for the CD3/T-cell correlation difference to the manuscript or supplement.
4. Remove or soften slightly dismissive wording such as "purportedly designed" when describing comparator methods.

These are minor edits. I do not think any remaining Reviewer #3 concern requires further major analysis.

(Remarks on code availability)
This is adequately addressed for publication.
The authors now point users to CRAN vignettes and provide benchmarking/figure-generation scripts. I did not rerun the full benchmarking pipeline, so this is not a full reproducibility audit. But the code/instruction issue raised by Reviewer #3 has been improved enough.

**Open Access** This Peer Review File is licensed under a Creative Commons Attribution 4.0 International License, which permits use, sharing, adaptation, distribution and reproduction in any medium or format, as long as you give appropriate credit to the original author(s) and the source, provide a link to the Creative Commons license, and indicate if changes were made. In cases where reviewers are anonymous, credit should be given to 'Anonymous Referee' and the source.
The images or other third party material in this Peer Review File are included in the article's Creative Commons license, unless indicated otherwise in a credit line to the material. If material is not included in the article's Creative Commons license and your intended use is not permitted by statutory regulation or exceeds the permitted use, you will need to obtain permission directly from the copyright holder.
To view a copy of this license, visit https://creativecommons.org/licenses/by/4.0/

**Reviewer's Comments:**

**Reviewer #1 (Remarks to the Author)**

The authors present cellGeometry, an R package for reference-based deconvolution of bulk RNA-Seq using single-cell atlases. The method employs "non-negative geometric deconvolution" (NGD), which performs vector projection of bulk samples against cell-type signature vectors, followed by spillover compensation with per-cell-type damping to ensure non-negative outputs. The manuscript emphasizes computational speed gains through matrix operations, benchmarks against MuSiC/DWLS/LinDeconSeq on pseudo-bulk simulations, and validates on whole blood and RA synovium cohorts. I believe that the method is a practical engineering contribution with clear speed advantages for atlas-scale references, but the core algorithmic novelty is overstated. NGD is essentially weighted least squares with a heuristic non-negativity constraint, and the manuscript does not convincingly establish that it represents a fundamentally new paradigm rather than an incremental variant of constrained regression with post-hoc regularization.

Major Concerns

1. The authors acknowledge in the Methods (lines 926-1018) that NGD has a "partial relationship to ordinary least squares" and is "equivalent to a weighted least squares solution." The core operations of vector projection via dot product, sphere scaling for equal gene weighting, spillover matrix as Gram matrix and compensation as matrix inversion, are all standard linear algebra from regression theory. The manuscript's geometric language (vectors, angles, projection, orthants) obscures rather than clarifies what is fundamentally $\beta = (X'X)^{-1}X'y$ with non-negativity post-processing.
The only distinctive component is variable compensation (tuning w_j per cell type to prevent negative outputs). However, this is presented as a heuristic optimization (golden section search to find w where $\min(\text{output})^2=0$) rather than derived from a principled objective. The manuscript states it is "related to, but distinct from, ridge regression" without formalizing exactly what NGD optimizes.

We thank the reviewer for their comments. In response we have:

i) Extensively revised the maths formulations and explained this in greater depth and clarity.
ii) We have added the objective optimisation formula in a new section in the Methods and explained what NGD optimises (namely the constraint all $B > 0$ whilst attempting to preserve projection).
iii) We have expanded the text to clarify differences and novelty between NGD and other regression methods, of which the closest equivalent is Tikhonov regularisation.
iv) As discussed below in point 2, we have benchmarked NGD against NNLS and L1/2 penalised regression (LASSO, ridge regression) and elastic net (glmnet) with non-negative constraints and shown that NGD is more accurate across the 4 scRNA-Seq simulations.

We believe the reviewer's comments regarding geometry are unfair. The geometric interpretation of different regression methods (OLS, ridge regression, LASSO, elastic net, SVM etc) is an accepted way of explaining how each method differs, as well as being the standard method for teaching students why some methods e.g. ridge and LASSO lead to biased estimators, where each type of bias has a purpose and use. That NGD has a mathematically neat geometric interpretation is an advantage.

By their arguments, the reviewer would presumably say that ridge regression has no purpose. It also fundamentally uses $\beta = (X'X)^{-1}X'y$, but adds a regularization parameter $\lambda$ which is simply a modified processing of $(X'X)^{-1}$, and the choice of $\lambda$ cannot be solved by an objective principle. $\lambda$ can only be solved either by arbitrary selection by the user, or by methods such as cross-validation.

Cross-validation still relies on minimising residual error in hold-out samples. We argue in **Fig. 5** that this is problematic where we show that the residuals of some genes in real-world analyses misbehave due to cells that are missing from the reference, or because of differences in gene expression due to inflammation or other pathogenic processes, e.g. infection, cancer etc. This is one of the main differences between our method and others. It was designed with the real-world user in mind, with a pragmatic solution which would give acceptable results even if SC and bulk datasets had issues relating to differences in experimental methodology.

The statement that NGD is the same as WLS is *only* true when compensation is fixed =1. In our view this is an unhelpful argument, which would be akin to arguing that ridge regression is equal to OLS when $\lambda =$

0, which it is. But as soon as $\lambda$ is not =0, then ridge regression becomes a useful advance upon OLS for specific problems such as collinearity. As soon as compensation **w** is not =1, NGD is clearly different from other methods.

Also, we differ from DWLS and MuSiC in that, both methods use an iterative process to solve the weights, both of which are comparable to IRLS (iteratively reweighted least squares). We use sphere scaling, so the weights are calculated in a single step. This deliberate lack of iteration is important as it provides a massive improvement in speed. We show that iteration is unnecessary as our method is accurate without it.

Deconvolution of bulk RNA-Seq as a mathematical problem can be reframed as an inverse problem in which the collinearity between predictors (cell types) causes the inverse mapping to operate as a high-pass filter with the undesirable tendency of amplifying noise.

NGD uses projection of each cell type signature to estimate the cell abundance. This has a huge advantage if the signature matrix has unknowable error in it compared to the bulk, which is often the case due to differences in sequencing chemistry between single-cell and bulk RNA-Seq. As NGD deconvolutes the bulk samples *together* rather than independently, NGD preserves linear proportionality between the cell-specific gene expression and the cell subclass abundance *across bulk samples*, which is desirable for end users. Hence even if there are problems (experimental noise/bias) between SC and bulk matrices, NGD will still yield results for each subclass that are sensible by being proportional to each cell-specific signature.

In contrast, the excessive application of the ill-conditioned inverse Gram matrix in other methods, will often lead to large and erroneous negative values. Even with a non-negative constraint, the most likely outcome is a poor overall solution. As we show multiple times in our manuscript, WCLS-based methods commonly conclude that particular cell types do not exist in the bulk, hence the solution commonly has cell types which are erroneously under-represented.

2. Given that NGD is essentially constrained least squares, the most relevant baselines are non-negative least squares (NNLS), NNLS with Tikhonov/ridge regularization, and other methods that explicitly enforce $\beta \geq 0$ during optimization rather than post-hoc. The manuscript only compares to MuSiC, DWLS, and LinDeconSeq (all W-CLS variants), plus NMF. NNLS is well-established, has fast implementations (e.g., nnls, glmnet with lower.limits=0), and directly solves the non-negative regression problem that NGD approximates with variable compensation. Without this comparison, it is unclear whether NGD's variable compensation offers accuracy improvements over simply using NNLS from the start.

Good suggestion. We have benchmarked NNLS using both the nnls and glmnet R packages across all 4 of the simulated datasets (**Supplementary Fig. 14**). Neither nnls nor glmnet with non-negative constraint over a range of alphas from 0 (ridge) to 1 (LASSO) performed as well.

3. All accuracy simulations use pseudo-bulk generated by Dirichlet sampling from the same scRNA-Seq dataset used to construct signatures (with arithmetic mean for simulations). This is an idealized setting where the reference perfectly matches the target. The noise experiments add perturbations but do not simulate domain shift where bulk comes from different chemistry, different cohort, or has cell types/states not in the reference.
The manuscript emphasizes robustness to "chemistry differences" but supports this primarily with noise transformations (log/sqrt/shift noise) rather than true cross-platform experiments.

We are not aware of any true cross-platform comparison data which is available for us to test. Datasets in which samples have been simultaneously subjected to scRNA-Seq and bulk are extremely rare due to cost and differences in sample processing. Single-cell requires either fresh or viably frozen tissue, or storage of nuclei. Bulk can be performed by taking biopsies and placing them immediately into RNA-later (or similar reagent) followed by homogenisation. These differences plus cost as well as the scarcity of human biopsies mean that cross-platform studies are exceptionally rare.

We have added to the manuscript the limitation that cross-platform studies would help to advance the deconvolution field (see also reply to Reviewer 2, point 3.2).

4. The manuscript describes iterative removal of genes with extreme Studentized residuals, presented as a diagnostic/correction mechanism. However, removing genes will always reduce residuals and SE. This does not guarantee improved deconvolution accuracy. In fact, removing true biology (e.g., interferon-response genes upregulated in disease) could bias estimates toward the reference state.
The blood example shows removal of granulocyte/RBC markers and interferon genes (interpreted as disease-relevant signal). But no quantitative validation demonstrates that iterative removal improves correlation with ground truth versus single-pass deconvolution.

Initially we found that increasing numbers of genes led to a sudden fall in accuracy with cellGeometry. This led us to discover that some genes had massive errors which showed up as outliers by regression methods looking at residual gene expression error. Therefore, we adapted cellGeometry to detect outlying genes and remove them in an automated manner. Users can set npass to any high number > 1 and the method will automatically stop after no more outliers are found. With each pass the outliers are removed until none are left. Outlier detection is very conservative by default (var.e = 4, Cook's distance = 1 or Studentized residuals = 10). But users have control and can alter the threshold for outlier removal, so that more outliers are removed at each pass. However, if the threshold is set to be excessively sensitive to outliers than large swathes of outliers could theoretically be removed at each pass and the process might not stop early. Hence we opted to give users the chance to control the stopping point in terms of number of passes. In practice, users can set npass = 5 and the algorithm will be conservative at removing genes and likely to only do 1-2 passes. We have clarified in the Methods the outlier thresholds and automation of npass.

We found that deconvolution of whole blood bulk RNA-seq using Cell Typist blood alone showed improved correlation with lymphocyte Coulter counter measurements with each pass of deconvolution (**Fig. R1** below). But overall, even with 4 passes, it showed worse correlation compared to using the merged gene signature which includes neutrophils (r = 0.53, P = 3.3e-05).

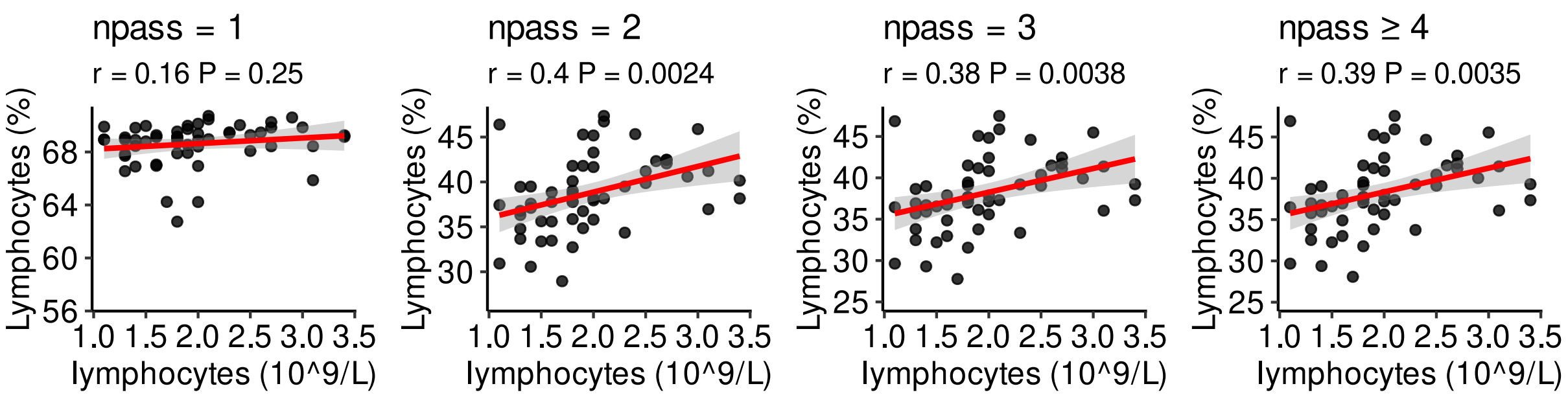


**Fig. R1.** Predicted lymphocyte percentages from deconvolution of whole blood bulk RNA-seq by cellGeometry had improved correlation with lymphocyte Coulter counter measurements with each pass of deconvolution after removal of outlying genes with extreme residuals.

5. The novel SE estimator based on cross-sample residual variance is interesting, but the manuscript only validates it via correlation with true error. Correlation indicates the SE tracks relative error magnitude but does not establish that the SE has correct absolute scale or frequentist calibration.

The SE is not a proportion of cells, it is reported in actual cell numbers and compared against error also in cell numbers, so it is on the correct scale. On the plots in **main Fig. 4f** the red lines show the identity line – this has been clarified in the legend. We have added Lin's CCC value to the Results text.

6. DWLS failed on 80.3% of R4RA synovium samples, reportedly due to quadratic programming breakdown. While this is a real usability limitation, the manuscript uses this to imply DWLS is an inferior method rather than simply unsuitable for this particular reference/bulk combination. DWLS performed comparably to cellGeometry in simulations.

We have provided a reprex script in the Github repo (https://github.com/EMR-bioinformatics/cellGeometry_manuscript/tree/main/04_Real_world_synovium/DWLS_reprex) which includes all the source code for the paper to allow users to test DWLS to show that with a real-world bulk RNA-seq from synovial biopsies in RA patients, that DWLS has an 80% failure rate in the quadratic

programming (QP) solution – the code abruptly exits with an error message for 80% of bulk samples: "Error in solve.QP(D, d, A, bzero) : constraints are inconsistent, no solution!".

See also reply to Reviewer 3, point 3: downsampling of the massive scRNA-Seq data (Tabula, Brain) enabled proper testing of DWLS. DWLS accuracy with the Brain dataset was poor (**Fig. 1c**, **Supplementary Table 1**).

See also detailed reply to Reviewer 3 point 12, where additional simulations with some subclasses set to 0 at random causes QP failure with DWLS with the same error message.

7. The manuscript emphasizes ability to deconvolute 18-461 cell subclasses, but validation against external measurements is limited to:
- Coulter counter: only neutrophils vs. lymphocytes (2 broad categories)
- Histology: CD3/CD20/CD68/CD138 (4 markers, semi-quantitative scoring)
Neither validates the fine-grained T/B cell subsets, monocyte subtypes, or fibroblast subpopulations that constitute most of cellGeometry's output. The correlation with Coulter/histology demonstrates consistency at the coarse level but cannot confirm accuracy for the detailed subclass estimates that differentiate this method.

Accepted, but unfortunately we do not have access to any publicly available datasets of peripheral blood that simultaneously include detailed FACS (with fine-grained B/T subsets) with matched bulk RNA-Seq.

Also, FACS is generally reported as % of cells within layered subpopulations - FACS is more accurate for estimating the proportion of cells within a particular gate. For example, lymphocytes are reported as % of PBMC; B cells are usually reported as % of lymphocytes; CD27+ memory B cells are reported as % of B cells etc. It is exceptionally rare for FACS data to be published in absolute cell count terms. FACS is often inaccurate at estimating absolute cell counts as variability in the layers of gating (e.g. PBMC > lymphocytes > live CD19+ B cells > CD27+IgD- memory cells) from one sample to another causes increased variability/noise in absolute cell counts. This would make it difficult to benchmark FACS results against fine-grained deconvoluted blood RNA-Seq.

The point we make in the Results is that other methods give estimates that do not make biological sense. For example, the T cell abundance with MuSiC is evidently much too low compared to known estimates of T cells in whole blood. T cells make up ~16% (range 6-31%) of white cells in whole blood (Melzer et al 2015, PMID: 25704947).

8. The Methods explicitly state the algorithm "assumes that each cell subtype is mutually distinct since it treats their dimensions as orthogonal". However, many biologically relevant cell states exist on continuums (Monocyte → macrophage differentiation; Naive → memory → effector T cell trajectories; Activation states within cell types). When applied to references with granular clustering (e.g., Tabula Sapiens with 461 subclasses), this assumption is systematically violated. The paper shows accuracy degrades with similar cell types but frames this as a limitation of the reference rather than the method. This is a fundamental constraint that affects interpretation.

The orthogonality of cell types is an initial assumption *only during the gene marker selection process*, when attempting to select optimal genes for the cell specific gene signature. The concept is to search for genes whose expression is perfect for each cell type, even in cell types whose trajectories show a continuum and overlap. If a particular cell state e.g. monocyte M1 or M2 has a specific gene which is only expressed in one of the cell states but in no other cell type, then this marker is perfectly specific for that cell type. However, the assumption of cell type orthogonality is explicitly not necessary during the deconvolution step. The point of the compensation process is that the basis vectors for each cell type do not need to be orthogonal. We have clarified this in the Methods.

9. All benchmark comparisons are presented as boxplots without formal statistical testing. Given that differences in RMSE/$R^2$ between methods are sometimes modest (e.g., cellGeometry vs. DWLS in simulations), it is unclear which differences are statistically significant vs. sampling variability.

Simulations were repeated x5 (except for brain which was repeated x3). We accept that in 3 out of 4 simple simulations (AMP, Cell Typist, Tabula), cellGeometry and DWLS are equally accurate, while statistical testing is unnecessary to prove that both cellGeometry and DWLS are clearly better than MuSiC

and LinDeconSeq in terms of accuracy – the standard error intervals for these comparisons clearly do not overlap. However, cellGeometry clearly outperforms DWLS with the more complicated Human Brain Atlas dataset. We have added new **Supplementary Table 1** showing overall accuracy results including Lin's CCC, RMSE and R-squared.

Where cellGeometry comes into its own and outperforms DWLS is in simulations with noise (**Fig. 4b**) and also the situation suggested by Reviewer 3 point 12, below where cell subclasses are randomly set to 0 (**Fig. 4c-d**, new **Supplementary Fig. 19**). This causes the QP solution to fail and also affects the accuracy of DWLS.

10. cellGeometry has numerous tunable parameters (noise filter ratio, expression filter, number of genes per subclass, compensation settings) that were presumably optimized. The baseline methods were run with "default settings". This asymmetry could favor cellGeometry.

All deconvolution methods in this domain have tunable parameters. For example, the damping parameters built into DWLS have been chosen by its authors based on their own experiments with datasets. Similarly, parameters in MuSiC and other methods (number of iterations, stopping points etc) have also been chosen and optimised by their authors.

We have provided a helpful function tune_deconv() within the package to allow users to see whether differences in parameters can help with simulated bulk data based with which users can test their own scRNA-Seq datasets. Testing of tune_deconv() shows that the tunable parameters listed by the Reviewer make only marginal improvements with simulated pseudo-bulk. The parameter that makes the biggest difference in simulations is the switch of the signature to an arithmetic mean. In the vignette we explain this and we have a table showing which parameters really matter and which settings are recommended for simulation versus real bulk RNA-Seq.

Minor Concerns

1. The abstract claims cellGeometry is "more accurate than existing methods" but benchmarks only include three methods from one comparative review. This is an overclaim given the >50 deconvolution methods in the literature.

We disagree that this is an overclaim. According to their comprehensive review, Nguyen et al (ref 6, PMID: 38619038) report that MuSiC, DWLS and LinDeconSeq were the 3 most accurate deconvolution methods out of a total of 53 methods benchmarked. As these methods have already been benchmarked by another major paper, this is not new and not publication worthy. We tested our method against these three leading methods and have shown that cellGeometry is consistently more accurate. More fundamentally the methods can be grouped by mathematical method and as we have already written, W-CLS previously performed best. We have additionally tested cellGeometry against other mathematical techniques including NMF, and in the revised manuscript we have also compared it against NNLS and NN elastic net (ridge/LASSO) as suggested by the reviewer.

2. Runtime comparisons lack hardware normalization. MuSiC cannot parallelize while cellGeometry uses 8 cores. Report single-core times for fair comparison, or normalize to core-hours.

Single-core times have been reported. The text has been clarified.

3. Gene selection by vector angle is intuitive but the number of genes selected per cell type, whether sets are disjoint/overlapping, and sensitivity to this parameter are not clearly reported.

The default number of genes per cell type (`nsubclass`) is 25, but overlaps in cell-specific genes between cell types are allowed and do occur. Users can alter the parameter `nsubclass`. The package includes a tune_deconv() function which allows users to test the effect of this on simulated pseudo-bulk datasets sampled from an scRNA-Seq references. In simple simulations, increasing nsubclass improves accuracy. This is not true once certain types of noise (esp. shift_noise) is added.

4. The "noise filter" (reducing low-level expression to zero) is described briefly but its effect is not ablated. What ratio threshold is used by default?

The default ratio is 0.25. The noise filter is not used during simulations as it modifies the gene signature matrix. Since the arithmetic mean is the BLUE (best linear unbiased estimator) of the basis vectors for each cell type, the noise filter affects this, leading to worse prediction of simulated pseudo-bulk, even though it improves the condition number (kappa), (**Fig. R2 below**). However, in real world data where there is unknown divergence between the scRNA-Seq data and the bulk data due to sequencing chemistry differences among other experimental differences, we find the noise filter reduces the condition number of the spillover matrix which helps to cope with noisy real world bulk data.

Also, see comments below for Reviewer 2, point 3.1 re high kappa values.

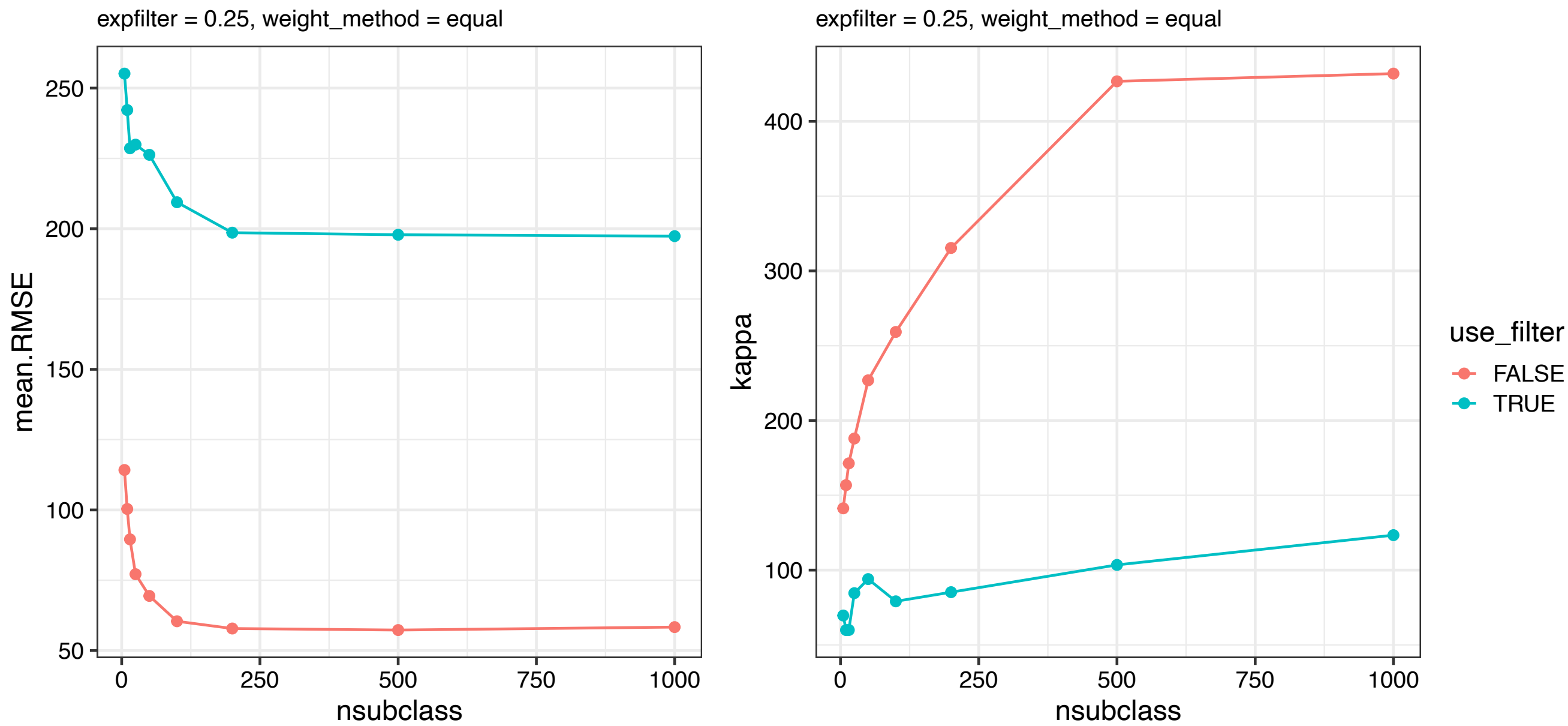


**Fig. R2.** Simulated pseudo-bulk data from Cell Typist was deconvoluted by cellGeometry using the tune_deconv() function to compare the effects of parameters, varying nsubclass (the number of genes per subclass in the signature) with or without the use of the noise filter (use_filter). This shows that the noise filter worsens accuracy (RMSE), but improves condition number of the spillover matrix (kappa).

5. Merging signatures via QQ mapping assumes distributions are similar. Provide sanity checks that merged signatures preserve expected marker relationships; potential distortions are not discussed.

The QQ mapping function in the package includes an S3 object plotting function which shows a QQ plot (double log axis) which can be used as a sanity check that the distributions are similar.

6. "Cell counts" vs "proportions" terminology is inconsistent. Most methods output proportions; cellGeometry outputs counts. Clarify the output scale and provide guidance on normalization for comparison.

cellGeometry outputs counts. The counts are subsequently converted to percentages by calculating the total number of cells in each sample and dividing cell counts by this.

7. The cosine similarity heatmap is mentioned but the actual heatmap is tiny/unreadable in the figure. Critical for interpreting deconvolution limits.

**Fig. 2b** has been updated to be more readable.

8. Histology validation shows correlations but acknowledges thin-section sampling limitations. Consider emphasizing this caveat more prominently when interpreting "accuracy."

The issue of thin-sections affecting histology accuracy has been acknowledged in the Discussion.

9. The claim that cellGeometry "redefines pathotypes" is potentially overstated. It refines pathotype classification using deconvolution-derived thresholds, but histology remains an independent modality.

We accept that in some clinical situations, rheumatologists will not have access to RNA-Seq and histology may remain important. However, we have discussed the pros and cons of using RNA rather than histology in the Discussion. Note that point 8 and 9 of the Reviewer conflict with each other: thin-sectioning of histology is a disadvantage compared to an RNA-based redefinition of the pathotypes.

10. The Methods state $\log_2$+1 transformation for signature generation but arithmetic mean of raw counts for deconvolution of simulated data. This inconsistency in scale should be more clearly justified.

This is an interesting point. $\text{Log}_2$+1 transformation is more robust against outliers. As scRNA-Seq is count based and extremely skewed even a single cell with high expression can generate a high value for the mean cell count if the arithmetic mean is used. One real example of this problem we encountered during this project is the *TRAV30* (T cell receptor alpha variable 30) gene in the AMP scRNA-Seq dataset. As its name suggests this is a T-cell specific gene. However in AMP1, a single fibroblast cell classified in the SC-F1 population expressed this gene at extremely high level (2218 counts). Raw data can be viewed here: https://singlecell.broadinstitute.org/single_cell/study/SCP279/amp-phase-1?genes=TRAV30&tab=distribution&distributionPlot=box&distributionPoints=all

Plots below show arithmetic mean [top] vs mean of log2(counts+1) [bottom] (**Fig. R3**):

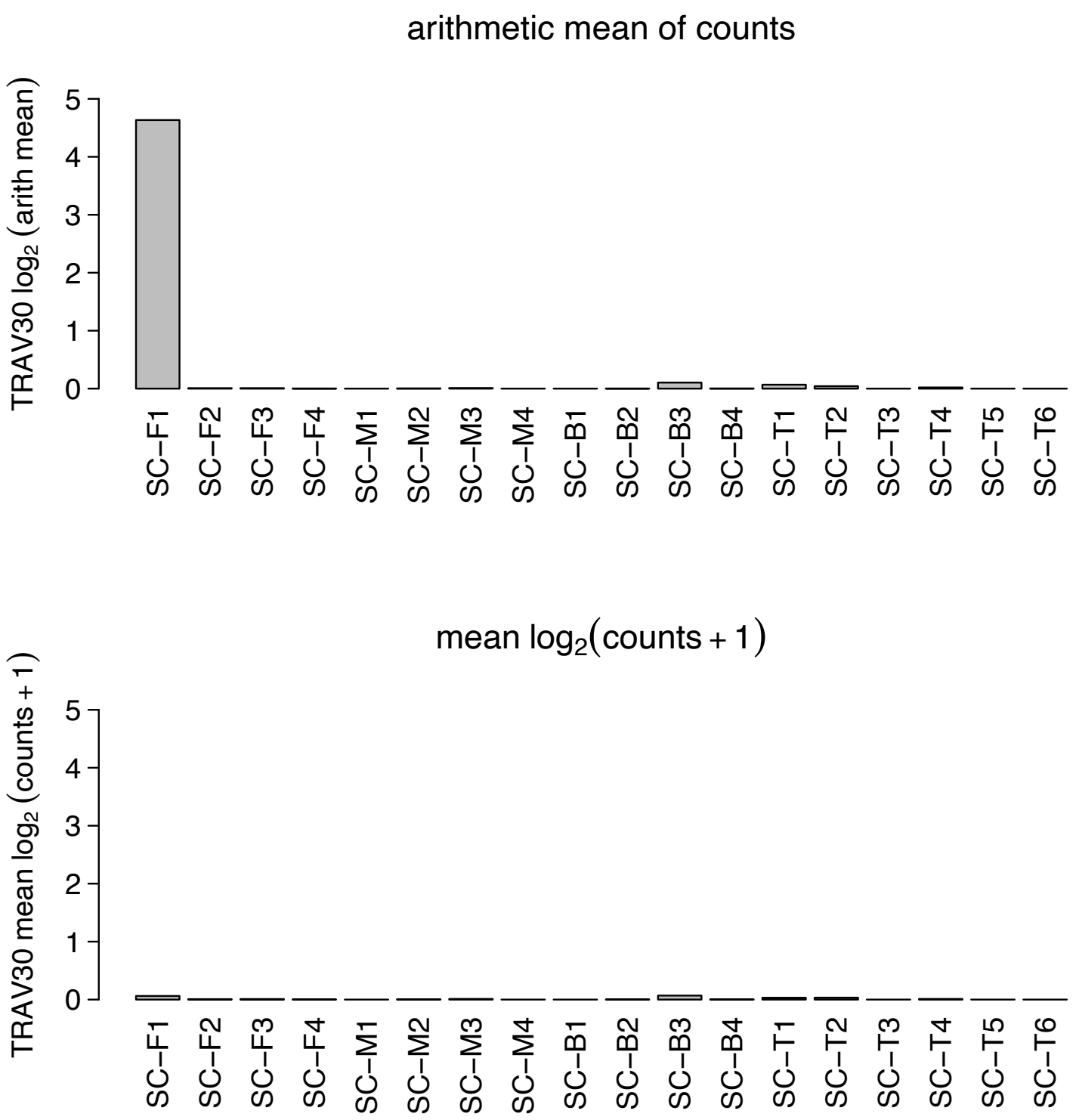


**Fig. R3.** *TRAV30* expression in AMP1 scRNA-Seq, comparing arithmetic mean versus mean of logged counts

During development, when we found that arithmetic mean of counts was best for the basis vectors for deconvolution of simulated pseudo-bulk, we tried using the same arithmetic of counts for the choice of signature genes. This gene *TRAV30* appeared as a highly specific gene for SC-F1 subclass which caused us to investigate it. The apparent high expression in the arithmetic mean is caused by a single erroneous F1 cell with extreme high counts. This shows how a more stable mean which is less sensitive to extreme outliers is required during signature gene selection. We have used the mean of $\log_2$ (counts+1) as the default mean function. However, we also provide the trimmed mean as an option with the package. This is another way to get the round the problem of outlying cells with erroneous massively elevated expression.

This illustrates nicely some of the core issues to be solved in deconvoluting bulk/scRNA-Seq. Arithmetic mean (of counts) is optimal for the basis vectors in pseudo-bulk simulations, but a different type of mean is necessary during gene signature selection to cope with extreme noise in count data.

(Remarks on code availability)

Yes. The package is well-documented and fully functional. Standard users can install and run the software following the provided vignettes. Full reproducibility of paper benchmarks requires the authors to make the benchmark repository public (https://github.com/EMR-bioinformatics/cellGeometry_manuscript/ is unavailable).

We apologise, this github repo, which we had originally intended to make public when we first submitted, has been made fully publicly available.

**Reviewer #2 (Remarks to the Author):**

1. Summary
This manuscript presents cellGeometry, a bulk RNA-seq deconvolution framework built from single-cell–derived signatures, framed through an explicit spillover/compensation view: a spillover matrix S captures cross-talk among reference cell types and the method uses compensation (conceptually S^{-1}) to correct spillover. A central technical contribution is variable compensation with non-negativity control, introducing a per-cell-type damping weight vector w in [0,1]^n to avoid negative abundance estimates while retaining much of the spillover correction. The paper also includes (i) a marker specificity/visualization scheme using unit-hypersphere scaling and vector angles, (ii) a residual-based uncertainty proxy (“var.e”) and related SE options, and (iii) a residual/outlier workflow that can highlight reference incompleteness and composition-adjusted biological programs (e.g., IFN-related residuals). The authors demonstrate utility in RA blood and RA synovium, including histology-grounded validation and a bulk-based redefinition of synovial pathotypes.

2. Strength
- Conceptual clarity and interpretability: The spillover/compensation framing makes the failure mode of closely related cell types concrete, and the non-negativity issue is handled via an explicit, inspectable mechanism (w-damped compensation).
- Operational robustness in difficult regimes: The manuscript documents settings where QP-based approaches can fail to return solutions under certain noise perturbations, while cellGeometry remains stable.
- Scalability is a genuine differentiator: The reported runtime improvements at thousands of samples and atlas-scale settings are substantial and align with the method’s matrix-operations design.
- Strong disease-relevant validation: In RA synovium, deconvolved abundances correlate with orthogonal histology markers and show improved biological plausibility relative to several baselines.
- Diagnostics beyond point estimates: The residual/outlier pipeline is practically useful: it flags missing cell types (e.g., granulocyte/RBC marker residuals when absent from reference) and can surface disease-state residual programs (e.g., IFN response) after accounting for composition.
- Uncertainty is addressed explicitly: The “var.e” approach is well-motivated and shown to correlate well with error in simulations, and the link to cell-type similarity is sensible.

3. Major Comments
3.1 Formalize the w-tuned variable compensation as an estimator, and add a stability diagnostic (conditioning): The non-negativity mechanism via w is the most distinctive algorithmic element. I recommend strengthening its statistical interpretability by (i) providing an explicit objective-function view (even approximate), and (ii) quantifying the bias/variance tradeoff relative to NNLS/QP baselines in high-collinearity regimes. In addition, because spillover correction is fundamentally limited by collinearity, I strongly suggest reporting a condition-number (or spectral) analysis of the spillover/Gram matrix (e.g., kappa(S)) across benchmarks and plotting it against deconvolution error. This would make the “not-resolvable subtype” regime explicit and give users a practical diagnostic for when subtype-level claims are not identifiable.

We have formalised the compensation mechanism as an estimator and added these formulae to the Methods (see also reply to Reviewer 1 point 1).

We have compared the cellGeometry method against NNLS using both the nnls R package and glmnet (see reply to Reviewer 1 point 2). NNLS does not perform well with this type of data. The reasons are not clear, but we suspect it may be partly due to the extreme skew of the data in count space. The deconvolution has to be performed in count space (as opposed to log space) since bulk counts per cell are only additive in count space.

We have added a kappa function which computes the condition number (kappa) of the spillover matrix for a deconvolution model. We have also added the ability to track kappa in the parameter tuning comparison tool tune_deconv(). This allows users to see how kappa is affected by changing deconvolution parameters (for example see reply to Reviewer 1 Minor #4 above). Although we have introduced pragmatic tools such as the noise filter which reduces the condition number (see Reviewer 1 minor #4), this tool is designed to be applied to real world bulk data. It makes simulated deconvolution results worse.

The classic method to improve the condition number (kappa) is to add a ridge parameter lambda. We have added this to the core package code, so that users that wish to add a lambda regularisation term to the spillover matrix can experiment with this. In our hands, in simulations we find that, similar to the noise filter, most values of lambda worsen the deconvolution of simulated pseudo-bulk (**Fig. R4** below) while small values provide very slight benefit which is offset by other parameters especially nsubclass. The figure below shows the effect of lambda on the whole dataset. We find that tuning lambda by cross-validation (CV) based on CV error does not help find the best value of lambda, which can only be found if the ground truth (the actual subclass cell counts) is known. Hence we do not advocate CV tuning of lambda within this framework as it results in only very modest improvement in accuracy.

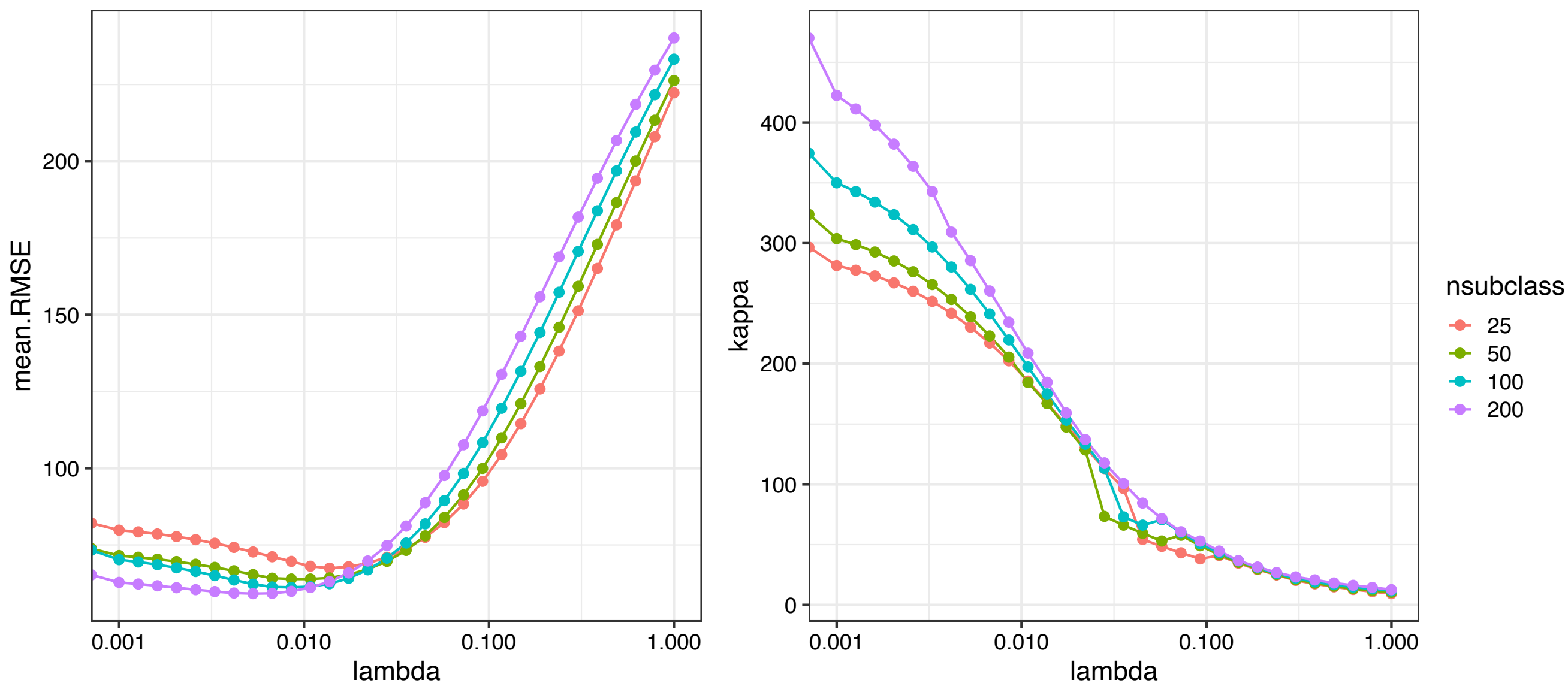


**Fig. R4.** Comparison of the effect of lambda ridge parameter on accuracy of deconvolution by cellGeometry and condition number (kappa) in simulated pseudo-bulk from the Cell Typist dataset. This shows that changing nsubclass (number of genes per cell type) has a stronger effect on accuracy than altering lambda. Although increasing lambda reduces kappa it does not necessarily improve deconvolution.

Our conclusion is that kappa is generally extremely high due to the underlying data being on the count scale (i.e. exponential) and not being Gaussian. Also, most users actively want to deconvolute subsets of related cells whose gene expression is relatively collinear, which also inflates kappa. cellGeometry does its best to cope with these eventualities. However, as suggested by the reviewer, we now provide a function to easily compute kappa within the package, and, practically speaking, users can then remove overlapping cell subclasses if they wish and see if kappa decreases (which it will).

3.2 Elevate domain shift to a primary benchmark: Noise-robustness and operational stability are important, and the RA synovium validation is compelling. However, the main failure mode in real deployments is reference misspecification/cross-study shift (chemistry, dissociation, donor effects, disease vs healthy). A

flagship “reference A -> bulk/pseudobulk B” evaluation (different cohort/lab/chemistry) would substantially strengthen the generality claim.

Real datasets, where the same samples have undergone cross-platform sequencing with both sc and bulk RNA-Seq, are extremely rare. We are not aware of any published studies that have examined this in depth. Also, it is technically impossible for the same cells to undergo cross-platform analysis – different portions of a tissue sample will have different cells that will vary in their inflammatory state, neighbouring cells, proximity to damaged tissue, blood vessels and other structures etc.

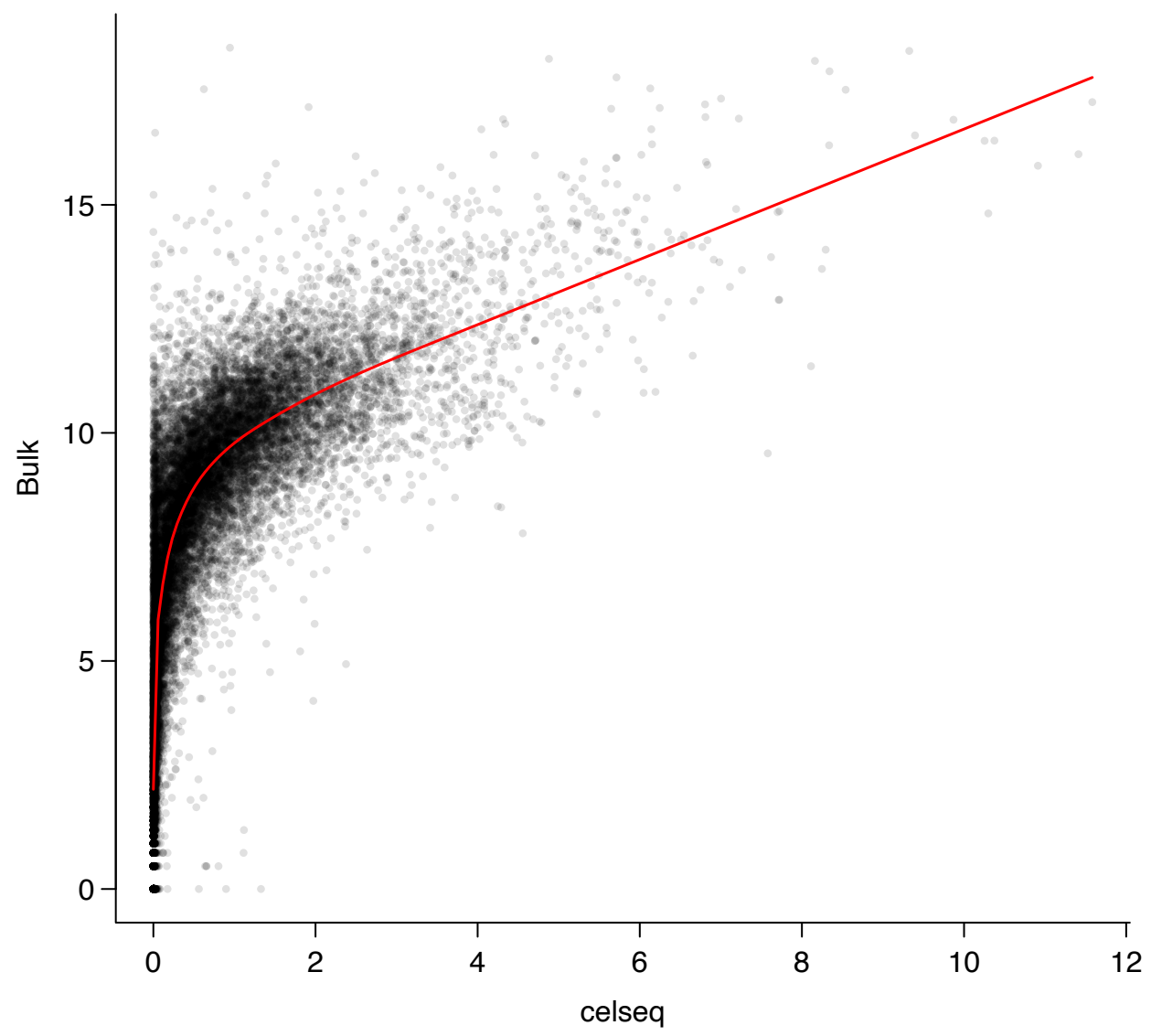


**Fig. R5**. Direct comparison of PEAC study RA synovium bulk RNA-seq vs single-cell RNA-Seq (celseq) from cells analysed in AMP phase 1. Each point represents a gene. Counts from the 2 bulk samples were summated then log2+1 transformed. The single cell data corresponding to both samples were first summated into a single pseudo-bulk sample, then log2+1 transformed.

The data above (**Fig. R5**) compares bulk RNA-Seq from 2 synovial biopsies from the PEAC study (Lewis et al 2019 Cell Reports, PMID: 31461658) which also underwent scRNA-Seq in the AMP phase 1 study (Zhang et al 2019, PMID: 31061532). These 2 samples were tracked to 1504 cells out of 10,099 cells in AMP with 17,988 genes overlapping between the sc and bulk RNA-Seq. (The remaining cells came from other non-PEAC biopsies, with no corresponding bulk sequencing.) The plot shows aggregated counts across cells from the celseq compared to bulk counts, both on a log2+1 scale. While there is a loose relationship between gene expression in the 2 chemistries, it is strongly non-linear and there is a considerable amount of noise at the individual gene level with log order differences. This is also evidence that bulk RNA-Seq is more sensitive than single-cell, since a large body of genes have reasonable bulk expression but close to zero celseq expression.

We originally tried to leverage this non-linear relationship within the cellGeometry package and early versions included a conversion from sc to bulk (and vice versa), so that deconvolution could take place in either SC or bulk sequencing dimensional space. However, in later versions we abandoned this conversion. It seemed to only work reasonably well for this single dataset (synovium bulk RNA-Seq being deconvoluted by AMP phase 1). It gave strange results with most other real datasets especially blood. Our conclusion is that the relationship between different bulk & scRNA-Seq sequencing chemistries may vary considerably from experiment to experiment. The above plot serves to highlight the problem that SC and bulk sequencing may have major experimental differences. Deconvolution algorithms must be robust to these types of real-world issues.

3.3 Validation metrics: add calibration-sensitive reporting (beyond correlation) and calibrate uncertainty. Some validation relies on correlation-style metrics or correlation-driven interpretation. Because Pearson correlation can mask systematic scale/shift bias in absolute abundance, I recommend adding Lin’s Concordance Correlation Coefficient (CCC) and identity-line calibration (slope/intercept or Bland–Altman) on key orthogonal validations. Separately, while var.e correlates with error in simulation, I would add

coverage-style calibration of intervals (nominal 90/95%), especially stratified by cell-type similarity, to make uncertainty actionable.

In nearly all plots and analyses, we are not using Pearson $r^2$, we have presented coefficient of determination $R^2$, (which runs from $-\infty$ to 1).

We now report a summary table detailing the RMSE, coefficient of determination $R^2$ and Lin's Concordance correlation coefficient (CCC) for the deconvolution of simulated pseudo-bulk datasets (**Supplementary Table 1**) and visualise the agreement between predicted and true cell percentages from deconvolution of simulated pseudo-bulk datasets as Bland-Altman plots (**Supplementary Fig. 6**).

CCC values are additionally reported in the manuscript for correlating between actual error and the deconvolution standard error for each cell subclass.

3.4 Make the residual "state after composition" layer a first-class analysis product.
The residual/outlier workflow is one of the most practically valuable contributions. I suggest formalizing this as a deliberate second layer ("composition-adjusted residual programs"), with explicit guidance on distinguishing technical mismatch (missing reference components) vs biological state shifts, and at least one replicated cohort example to demonstrate stability.

We have added S3 class functions for `residuals()`, `rstandard()` and `rstudent()` to the package which can be applied to deconvolution model objects. This enables users to extract raw, standardized and Studentized residuals. The residuals.deconv() S3 function also permits users to obtain raw residuals for all genes, not only those that are subsetted into the cell-specific gene signature matrix. We also provide functions for users to generate outlier metrics such as Cook's distance on the fitted models, so that users can examine these statistical results to help them decide whether genes are outliers. We have added a plot_residuals() function to allow users to visualise residuals similar to plots shown in the paper.

3.5 Pathotype redefinition: justify thresholds and assess stability.
The bulk-based RA synovium pathotype redefinition is appealing, but hard thresholds (e.g., B cells > 30; macrophages > 300) raise concerns about protocol dependence and biopsy cellularity. A sensitivity analysis over thresholds (and/or normalization considerations) would strengthen the translational story.

We accept that the cellular thresholds could be perceived as being arbitrary, but they have been set based on our results from 3 separate international multi-centre biopsy cohorts in discussion with pathologists at our centre.

The original histology cell count cut-offs were also determined by expert histopathologist opinion rather than by data driven cut-offs. The original histopathology cut-off between B-cell rich (= lymphoid pathotype) and B-cell poor (= fibroid & myeloid pathotypes) biopsies, had a cut-off of <=20 B-cells for B-cell poor and >= 21 cells for B-cell rich (Rivellese F et al 2020, PMID: 31785084). However, this was on the basis of a single 3 um section rather than a whole volume of biopsy. No such cell number cut-off was made for macrophages as these can be scattered throughout the section. It was based on aggregated macrophage groupings which were assessed by eye. Hence even in the original histological pathotype definition the macrophage number was arbitrary and affected by spatial localisation information such as aggregation.

We used the rpart package to generate decision trees to predict the histology pathotypes based on the cellGeometry results from **main Fig. 6d**. This analysis was undertaken by sampling 90% of the data (divided into 10-folds, 1 fold removed each time), with 15 repeats of the 9/10-folds. On average the B cell threshold was 25 and the macrophage cell threshold was 388 (**Supplementary Fig. 26a**). We have now updated the figures on the pathotype redefinition with these data-driven thresholds (**main Fig. 6d, Supplementary Fig. 26-27**).

4. Minor Comments
- Provide a compact algorithm box that standardizes notation across Y, G, H, S, compensation, and w, and clarifies what is computed in count vs log space (since this impacts additivity and interpretation).
- The vector-angle marker ranking is clear and useful, but should be explicitly positioned as cell-type specificity rather than state-program discovery (to avoid over-interpretation in continuous-state settings).

- Provide recommended defaults and stopping criteria for iterative extreme-residual removal, and clarify how it interacts with compensation tuning.

We have added a compact algorithm box to make it easier to understand the algorithm.

The iterative extreme-residual removal process is designed to automatically stop after a period of time with the current defaults, which default to only removing the most extreme outliers. This means that the iterative `npass` system for removing outlier genes can be set at a high value and the algorithm will tend to stop automatically once no further outliers are detected. This usually only takes either 0 or 1 pass in most simulated situations, unless an orchestrated simulation where outlying genes are deliberately introduced such as using the shift_noise function with a large noise standard deviation is employed. In our experience with real-world datasets, once npass is >=4 the algorithm typically automatically stops as it cannot find any further outliers. The exception to this is that if the outlier threshold is reduced markedly then the algorithm may go on removing outliers forever, which is why npass has a fixed value. Most users can just set npass = 4 and that seems to be sufficient in all real-world scenarios which we have tested. We have added a paragraph “Multipass removal of outlier genes…” to the Methods to give details of this method including recommended defaults.

- Make benchmarking conditions maximally explicit (hardware, parallelization, preprocessing parity) given the very large runtime differences reported.

We have added detail to the Methods and made single vs multi-core runtimes clearer.

5. Overall
This is a strong, well-motivated, and practically impactful methods manuscript. The combination of (i) explicit spillover/compensation geometry, (ii) a pragmatic non-negativity mechanism, (iii) strong RA synovium validation with orthogonal support, (iv) a genuinely useful residual diagnostic layer, and (v) standout scalability makes it compelling as more than an incremental engineering exercise. The main upgrades that would most materially elevate the manuscript’s impact and perceived rigor are: (1) an objective-function and bias/stability characterization of w-tuned compensation plus a conditioning diagnostic, (2) a headline domain-shift benchmark, and (3) calibration-sensitive validation metrics (CCC + identity calibration) alongside calibrated uncertainty. With these additions, I would be comfortable advocating this manuscript for Nature Comms.

Reviewer #2 (Remarks on code availability):

- Code availability appears strong and reproducible, with extensive documentation and vignettes
- I was able install the package out of the box and was able to explore a few vignettes without any issue

**Reviewer #3 (Remarks to the Author)**

The authors present a deconvolution method called cellGeometry that uses matrix operations for a faster deconvolution. The authors test the method’s performance in simulated bulks and real bulks, and compare results to other well-performing methods, across different tissue types. CellGeometry consistently is shown to be faster and outperform other methods. The method’s implementation is modular and is offered as an R package for easy use. The authors are very thorough in their benchmarking and offer all information in figures.

Comments:

1- In the section “Ability to merge single-cell reference signatures” and others, the authors describe cellGeometry as having the “unique ability” to merge gene signatures from different scRNA-seq datasets. Then the authors say MuSiC does not have the feature of merging single cell clusters, and omit comparison analysis because of this. Can you elaborate on what this means? MuSiC is specifically for multiple subjects/datasets, and simply changing cell type names could help create the desired gene signature, and adding batch or patient ID will likely increase accuracy if not currently implemented. I would suggest the authors address this in the revised version of the manuscript.

We are specifically referring to merging of 2 distinct scRNA-Seq datasets. While this is possible manually, it requires aligning the rows of genes and binding columns of the 2 raw SC count matrices together, then concatenating the SC metadata, and adding a column for batch. However, that compute process is not trivial when the count matrix and metadata run into 30 GB file sizes, where the finalised merged objects need to be saved to disc. MuSiC has no way to specify multiple scRNA-Seq dataset objects as input.

In our simulation of the brain dataset the simulated pseudo-bulk is generated by sampling from both the neuronal and non-neuronal SC datasets and then summing the resulting bulk gene expression. We show that analysis of the neuronal and non-neuronal datasets by MuSiC is i) extremely time consuming and ii) inaccurate.

cellGeometry takes a different view. It merges only the *signature* matrices, not the raw single-cell count matrices. This is considerably more convenient for users and avoids manipulations of massive on-disc matrices. If the signature matrix may have a slightly different scale (this varies depending on number of cycles of PCR etc during library prep), the distributions of the signature matrices are aligned using QQ mapping before merging. With the brain atlas datasets, QQ mapping is not required as the 2 SC datasets were generated by the same lab using the same scRNA-Seq methodology. In this situation cellGeometry is able to deconvolute simulated bulk brain RNA-Seq containing both neuronal and non-neuronal cells without degradation in accuracy.

2- I suggest the authors corroborate that each method is used as intended. For example, like mentioned above, MuSiC is meant to have multiple subjects, so patient/batch information should be passed to the model. Furthermore, the authors later write some cells were used with only 4 cells in the single-cell reference. This is contrary to the specifications of most methods that exclude analyses when there are less than 10 cell types. For example, MuSiC's reliable estimation depends on sufficient sampling to estimate cross-subject means and variances. The DWLS failures are due to the method not being used as expected mathematically (too few cells per example), not a method failure but a user error. If cellGeometry is able to deconvolve accurately with such a low number of cells per cell type, then that is a big advantage of the method. However, the other methods should be used as intended for fair comaprison. I would suggest rerunning the analyses with this information, or address this by adding an analysis testing number of cells in reference vs. performance. Cell types can be merged for usable cell type numbers if needed. Outside of showcasing the ability to deconvolve very granular cell types, I don't see a need to use cell types with less than 25-75 cell examples across multiple datasets, especially since at that level of granularity most real bulk samples will not have all states of a particular cell type, and reporting all of them in real bulk is concerning (detecting "all 18 subclasses in the synovium" could likely be cellGeometry error rather than true presence of cells).

cellGeometry removes cell subclasses with <10 cells in the scRNA-Seq by default since we believe there is insufficient information if fewer than 10 cells are present. This cutoff can be controlled by users through a parameter setting.

The authors of DWLS state that one of the rationales of DWLS was to overcome rare cell population deconvolution, but they do not specify how rare. Both MuSiC, DWLS and LinDeconSeq give no specific instructions for removing cell subclasses below a particular threshold. MuSiC2 was designed with 2 bulk tissue datasets in mind (control vs disease) to be able to handle the situation where some cell subclasses might be rare and only present in one bulk dataset. However, we could not find a way to use this method as we did not have matching control vs disease bulk datasets and had no easy way to simulate this. MuSiC2 removes cell subclasses where the mean proportion of cells across all samples in the bulk data is < 10% of the cells as the default (prop_r = 0.1). This means it is mainly designed only to detect much more common cells and will remove rare cells in bulk by default. MuSiC1 & 2 do not specify a cutoff for the number of cells in the scRNA-Seq dataset.

We re-ran MuSiC, LinDeconSeq and DWLS for the deconvolution of real-world whole blood bulk RNA-seq whilst removing single-cell clusters with <10 cells in the reference. This hardly altered their deconvolution results (**Table R1** below) and the correlation between predicted lymphocyte percentages and lymphocyte Coulter Counter measurements did not improve (**Fig. R7** below).

In the AMP synovium dataset, the minimum number of cells per subclass was 48. We considered all of these subclasses to be important and functional, as described in the AMP paper (Zhang F 2019, Nature immunology, PMID: 31061532). As we believe from earlier histological studies that we are likely to find all

of these cell types within inflamed rheumatoid arthritis synovial tissue (albeit not in all patients all of the time), we did not remove any of these subclasses.

We provide the bulk synovium RNA-Seq dataset for users to test out DWLS for themselves.

| Cluster | CellGeometry | MuSiC | DWLS | LinDeconSeq |
|---|---|---|---|---|
| **Memory B** | 2.02 (0.00 - 4.13) | 0.00 (0.00 - 0.00) | 0.59 (0.00 - 2.52) | 3.12 (0.00 - 11.4) |
| **Naive B** | 1.68 (0.00 - 5.15) | 0.00 (0.00 - 0.00) | 1.11 (0.00 - 4.38) | 3.78 (0.00 - 13.7) |
| **Plasma** | 1.01 (0.00 - 2.21) | 0.00 (0.00 - 0.00) | 0.00 (0.00 - 0.10) | 0.35 (0.00 - 2.25) |
| **DC** | 1.96 (0.00 - 5.19) | 0.00 (0.00 - 0.00) | 0.00 (0.00 - 0.00) | 0.35 (0.00 - 2.77) |
| **DC2** | 2.42 (0.00 - 3.80) | 0.00 (0.00 - 0.01) | 0.00 (0.00 - 0.00) | 3.76 (0.00 - 9.42) |
| **pDC** | 1.09 (0.00 - 2.11) | 0.00 (0.00 - 0.00) | 0.01 (0.00 - 0.38) | 0.25 (0.00 - 0.57) |
| **Classical monocytes** | 9.36 (0.00 - 20.2) | 39.8 (14.5 - 65.2) | 0.95 (0.00 - 7.12) | 30.7 (13.9 - 53.3) |
| **Non-classical monocytes** | 10.5 (6.2 - 18.5) | 12.8 (1.91 - 29.9) | 30.1 (3.25 - 82.5) | 0.08 (0.00 - 3.07) |
| **Macrophages** | 1.11 (0.00 - 1.79) | 0.00 (0.00 - 0.00) | 2.83 (0.00 - 12.7) | 0.00 (0.00 - 0.00) |
| **Follicular helper T** | 2.39 (0.00 - 4.98) | ND*** | 0.61 (0.00 - 3.80) | 0.00 (0.00 - 0.00) |
| **MAIT** | 1.96 (0.32 - 9.78) | 0.00 (0.00 - 0.00) | 0.85 (0.00 - 4.71) | 4.22 (0.88 - 7.94) |
| **Regulatory T** | 4.18 (1.79 - 13.1) | 0.00 (0.00 - 0.10) | 0.52 (0.00 - 2.01) | 1.67 (0.00 - 10.1) |
| **Tcm/Naive cytotoxic T** | 4.80 (0.00 - 7.42) | 0.00 (0.00 - 0.00) | 0.00 (0.00 - 0.00) | 0.00 (0.00 - 0.00) |
| **Tcm/Naive helper T** | 3.16 (0.00 - 7.07) | 0.00 (0.00 - 0.00) | 0.00 (0.00 - 0.00) | 19.4 (0.00 - 32.5) |
| **Tem/Effector cytotoxic T** | 1.66 (0.63 - 2.75) | ND*** | 0.30 (0.00 - 2.62) | 0.00 (0.00 - 0.00) |
| **Tem/Effector helper T** | 5.71 (0.16 - 8.86) | 0.09 (0.00 - 0.26) | 0.86 (0.00 - 5.23) | 7.19 (0.00 - 15.63) |
| **Cytotoxic T** | ND* | 0.00 (0.00 - 0.00) | 1.91 (0.00 - 8.82) | 15.3 (6.37 - 28.1) |
| **Helper T** | ND* | 0.00 (0.00 - 0.00) | 0.00 (0.00 - 0.00) | 0.74 (0.00 - 19.5) |
| **Gamma delta T** | ND** | ND*** | ND** | ND** |
| **Cycling T** | 0.67 (0.00 - 1.42) | 0.00 (0.00 - 0.01) | 0.00 (0.00 - 0.02) | 4.58 (0.00 - 10.9) |
| **CD16+ NK** | 4.27 (0.43 - 11.0) | 14.4 (0.00 - 35.0) | 0.19 (0.00 - 3.56) | 0.05 (0.00 - 3.55) |
| **CD16- NK** | 1.61 (0.00 - 2.45) | 0.00 (0.00 - 0.00) | 1.26 (0.00 - 4.26) | 0.12 (0.00 - 3.39) |
| **NK** | 1.89 (0.00 - 3.15) | 9.44 (0.00 - 32.8) | 0.00 (0.00 - 0.00) | 2.14 (0.00 - 5.15) |
| **ILC** | 2.45 (0.07 - 7.49) | 0.00 (0.00 - 0.00) | 0.00 (0.00 - 0.00) | 0.00 (0.00 - 0.05) |
| **Mast** | 2.87 (0.00 - 4.32) | 0.00 (0.00 - 0.21) | 0.30 (0.00 - 1.55) | 1.09 (0.00 - 8.09) |
| **Basophil** | 1.37 (0.48 - 2.21) | | | |
| **Neutrophil** | 23.1 (10.4 - 37.6) | | | |
| **Early MK** | 4.81 (1.23 - 20.5) | 23.6 (14.3 - 36.7) | 57.2 (0.78 - 93.6) | 0.95 (0.19 - 2.95) |
| **MEMP** | ND** | ND** | ND** | ND** |
| **Promyelocytes** | ND** | ND*** | ND** | ND** |
| **HSC/MPP** | 0.53 (0.00 - 1.28) | 0.00 (0.00 - 0.00) | 0.00 (0.00 - 0.00) | 0.00 (0.00 - 0.00) |
| **GMP** | 1.38 (0.00 - 4.12) | ND*** | 0.45 (0.00 - 4.53) | 0.07 (0.00 - 0.54) |

**Table R1.** Cell subclass percentages from deconvolution of rheumatoid arthritis whole blood bulk RNA-seq using different deconvolution methods. Deconvolution results using cellGeometry is unchanged but MuSiC, DWLS and LinDeconSeq was undertaken with the removal of cell subclasses that have less than 10 cells in the single cell dataset, which are highlighted in yellow. Cell percentages are shown as mean and range, limited to 2 decimal places. Cell subclasses with outputs of zero across all samples are highlighted in red. * These subclasses were removed from the Cell Typist blood signature due to their similarity with other closely related CD4 and CD8 T cell subset clusters. ** Gene signatures were not generated for these subclasses because of too few (<10) cell numbers in the scRNA-Seq. *** These subclasses were not outputted from the deconvolution algorithm

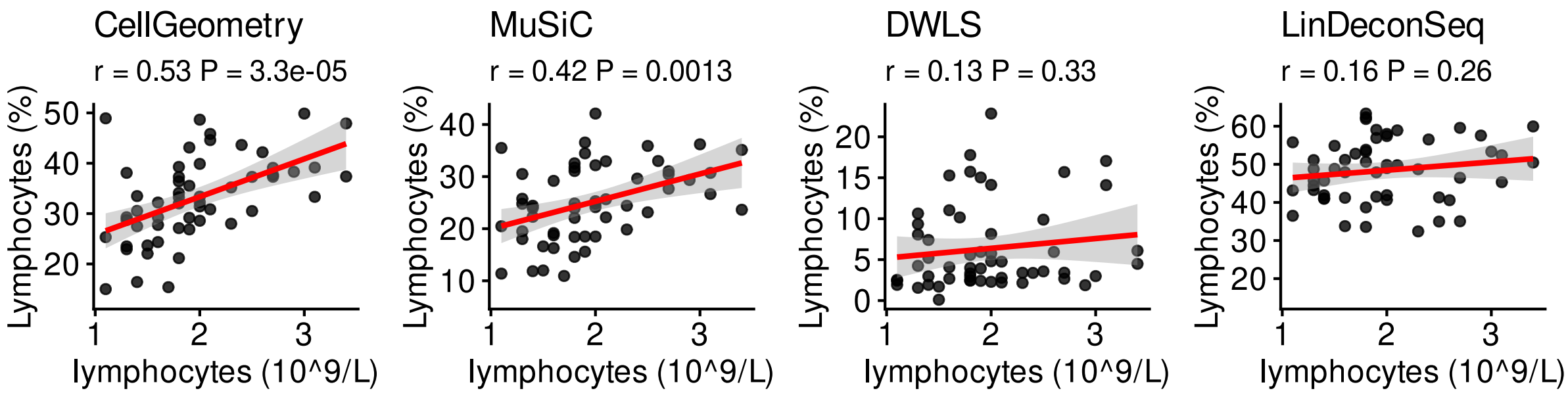

**Fig. R7.** The correlation between predicted total lymphocyte proportions and lymphocyte Coulter Counter measurements for MuSiC, DWLS and LinDeconSeq did not significantly improve when removing cell subclasses with too few cell numbers (<10) in the reference scRNA-seq

3- Related to the above comment, there is research showing that increasing the number of cells per cell type, or number of cells in the reference, does not increase deconvolution accuracy in some methods (accuracy plateaus past a certain point). The authors sometimes omit analyses based on R running out of memory, etc. However, it could be that including less cells per cell type (controlled in the proper manner) could 1. Make those analyses possible in R, particularly those that failed DWLS, and 2. Would increase accuracy of all methods. I would suggest the authors address this in the revised version of the manuscript.

To overcome the matrix limit of $2^{31}$ elements, we restricted the maximum number of cells to a fixed amount and sampled for the cell subclasses randomly to stay within the $2^{31}$ limit. This reduced each of the 1-3 million cell scRNA-Seq datasets to around 20-30,000 cells. The subsetted Human Brain Cell Atlas and Tabula Sapiens datasets were used to assess deconvolution by DWLS and LinDeconSeq.

In spite of subsetting the cells, LinDeconSeq was unable to deconvolute both Human Brain Cell Atlas (neuron) and Tabula Sapiens. It failed at the signature generation step. Full details and a reprex script have been provided on the Github repo: https://github.com/EMR-bioinformatics/cellGeometry_manuscript/tree/main/02_Stimulated_benchmarking/No_noise/LinDeconSeq_reprex

Even with the scRNA-Seq subset to 20-30,000 cells, DWLS took 54 hours to generate the gene signature for Tabula Sapiens. Although DWLS had high accuracy for deconvoluting Tabula Sapiens, DWLS could only consider neuron and non-neuronal cells separately and so did not perform as well (**main Fig. 1c** [revised]).

Human Brain Cell Atlas non-neuronal simulated data was deconvoluted using DWLS and LinDeconSeq where both did not perform well and were significantly worse than cellGeometry (**Supplementary Figure 17**).

4- The authors have no differentiation of single cell and single nucleus RNA-seq datasets within the Human Brain Atlas (could be others). It is commonly reported that neurons and other cell populations are not able to be observed in single nucleus datasets, so I assume the datasets used contain both, combined Single cell references and single nucleus references can impact deconvolution in most methods, and I would suggest the authors consider this.

The Human Brain Cell Atlas is single-nucleus RNA-seq, whereas the other datasets (AMP rheumatoid arthritis synovium, Cell Typist and Tabula Sapiens) are single cell. We have clarified this in the text.

5- The use of results to adjust gene signatures and improve deconvolution is very interesting. Out of curiosity, have the authors applied this to other deconvolution methods as well? i.e., would DWLS also improve accuracy with multiple passes after gene filtering? Could other methods also benefit?

Using the genes that were filtered out by cellGeometry for the deconvolution of whole blood RNA-seq from the PEAC clinical study using the Cell Typist blood dataset (as described in **main Fig. 5a-c**), we have removed them from the DWLS signature and from the Cell Typist blood reference for MuSiC. MuSiC is a one-step deconvolution process so the signature cannot be directly intercepted and adjusted accordingly. It was noted that LinDeconSeq gene signature already did not contain the genes filtered out by cellGeometry.

With these adjustments, whole blood RNA-seq deconvolution was rerun for MuSiC and DWLS (**Fig. R8 a-b** below) where we generate same comparisons as main **Fig. 5e & g** (for ease of comparison **Fig. R8 c-d** below). The correlation of lymphocyte percentages with Coulter counter measurements improved slightly, but was still not as good as cellGeometry (r = 0.53, p = 3.3e-05). We have noted in the manuscript text that gene filtering of the gene signatures in other deconvolution methods was performed, but only partially improved accuracy. Both MuSiC and DWLS still overestimated monocytes and underestimated T cells.

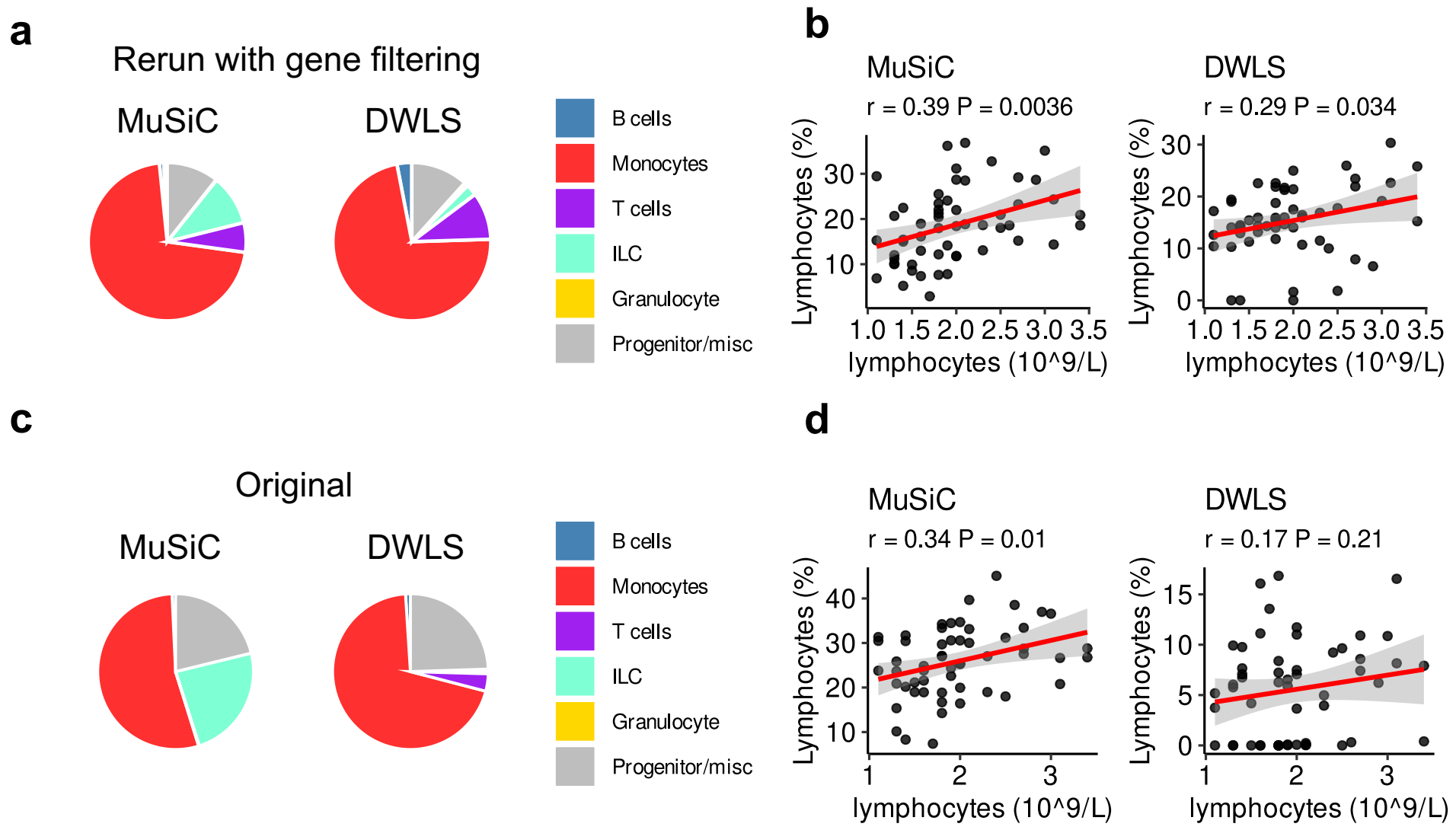


**Fig. R8.** The deconvolution of whole blood RNA-seq with gene filtering of the gene signatures (a-b) did not substantially improve its accuracy compared to the original deconvolution analysis (c-d) for MuSiC and DWLS

6- There are many supplementary figures showing real vs. predicted proportions, and some of these take over multiple pages. The manuscript would be more digestible if the authors made the figures only be one page at the most, and merged some of the panels to minimize the number of supplemental figures. You can try multiple cell types in one plot, with different shapes or colors. Also, it is concerning that some of the values in the supplementary figures showing predicted vs. "real" cell type proportions go past the 1.2 tick mark. The assumption is proportions sum to 1, so cell types can not have a proportion higher than 1, specially in mixture. I would kindly ask the authors to explain why values go past 1 in real proportions.

These were cell subclass percentages rather than fractional proportions which add up to 1. The legend has been amended to clarify. The correlation plots for Tabula Sapiens and the Human Brain Cell Atlas simulated data have been consolidated into single plots for each deconvolution method (**Supplementary Fig. 9-11**).

7- It seems from Fig. 6B that MuSiC best aligns with the cell populations expected in bulk RNa-seq samples, even if cellGeometry detects more cell types (which is not what one would expect in bulk RNa-seq). The manuscript would be strengthened if the authors addressed this.

This is addressed in **Fig. 6c** where the MuSiC estimated T cells do not correlate with CD3 histology.

8- In Supp. Fig. 18b, the Gaussian noise increase causes no change in Rsq. This is very contrary to what is usually observed in models like DWLS, MuSiC. Do the authors have an explanation for seeing no change in performance?

There are 2 main reasons:
i) Since the RNA data is on count scale, Gaussian noise barely touches genes with high expression e.g. whose counts run into 10^5 or more. As these genes are untouched and often more highly leveraged in deconvolution methods, highly expressed genes provide full information for the algorithm.
ii) Gaussian noise does not change Rsq unless the noise is very, very large because the noise is unbiased. So on a population level most methods use many genes (equivalent to large n) and if each gene

is affected equally by noise, then if the deconvolution method is BLUE and so based on averaging inference from many genes it is not affected much by Gaussian noise. Noise methods with intrinsic bias will more heavily affect these systems. Non-Gaussian noise (log, sqrt) will be upwardly biased once converted back to count scale. Because shift_noise selects genes at random, this causes bias in whole genes. If multiple genes which are cell-specific are shifted this will alter the deconvolution in a biased, non-symmetric way, so methods are more sensitive to this.

And could the authors also explain what are the values in the x axes of these figures (Gaussian noise from 0-500)?

The $x$ axis refers to the standard deviation (SD) of noise, using rnorm() but on the scale which the noise is applied. For example in log noise, bulk data is transformed by log2+1 then noise is added via rnorm(), then the data is transformed back to counts. This has been clarified in the Figure legend.

It would be worth shortening some of the figure legends by removing redundant information to help make the axes/figures more interpretable, keeping only the needed information. If something is reported as results, there is no need to report again in figure legends.

We have made the legends more concise.

9- I recommend the authors be more cautious about using the words “novel” or “unique” throughout the manuscript. For example, using word novel when describing the variable compensation nnmf and also describing var.e SE measure.

We have reduced the use of the words “novel” and “unique” as suggested.

10- The authors show in Fig. 5 the decreasing residuals with more passes. I would kindly suggest adding a label to what the red values are vs. black values in the Fig. 5a plots. And can you make it clear how exactly the outlier genes are classified as outlier? a threshold?

We have added a paragraph subheaded “Multipass removal of outlier genes based on extreme residual gene expression” to the Methods to explain the multipass removal of outlier genes in more detail. We have added to the **Fig. 5** legend to explain the different coloured points in these plots. Each point represents the residual gene expression per gene per bulk sample. Outlying genes are flagged in red, and non-outlier genes are in black. The cut-off for a gene being an outlier is deliberately set to be high by default so that it is only triggered by extreme outliers, but this can be user controlled.

11- In Fig. 6c, the authors show the correlation of marker expression vs. calculated cell abundances/proportions for 4 cell groups. Is there a reason this analysis was done with these 4 groups rather than the same 4 groups explored in Fig. 6b? Both panels would be strengthened by using the same 4 groups. Also, the correlation coefficients are very close in some cases, making the results comparable, and this is not addressed in results (only cellGeometry outperformance is mentioned).

The major cell groups shown in **Fig. 6b** are the overarching 4 cell groups from the single-cell reference. Unfortunately, fibroblast histology had not been generated for the synovial biopsy histology analysis in the RA cohorts, which is why there is no fibroblast marker immunohistochemistry (IHC) semi-quantitative score in **Fig. 6c**. Fibroblast staining was not part of the IHC panel in these RCT. Only macrophages (CD68), T cells (CD3), B cells (CD20), plasma cells (CD138) were stained.

We have amended the text to be more circumspect regarding the close correlation coefficients across the methods for certain cell types.

12- After reviewing the manuscript, the question remains of whether the very granular identified cell populations (multiple subsets of T cells, etc.) truly represent real cells within real bulk. The manuscript would be strengthened by showing cellGeometry and other methods with negative controls: “if a pseudo-bulk does NOT include X granular cell type/state, is the deconvolution method still predicting it?”.

This is a very helpful suggestion. Using the AMP scRNA-Seq dataset, we generated simulated pseudo-bulk data where varying proportions (0%, 5%, 10%, 20% and 50%) of the cell subclasses were randomly set to zero for each sample. We used this to benchmark cellGeometry against MuSiC, DWLS and

LinDeconSeq, with 5 repetitions. The high accuracy of cellGeometry remained consistent irrespective of cell absence (**Main Fig. 4c; Supplementary Fig. 19**), whereas DWLS accuracy worsened with increasing proportion of cell subclasses that were set to zero and notably the quadratic programming failure rate of DWLS increased in proportion to the fraction of absent cell subclasses (**Main Fig. 4d**). Both MuSiC and LinDeconSeq performed less well than cellGeometry, and frequently erroneously predicted the presence of absent cell subclasses. We have included this new data in the manuscript.

Minor:

- Consistency in paragraph indentation, italics genes, and acronym introductions.
- Sentences in formal writing typically don't start with numbers or lower case letters.
- References for deconvolution methods in introduction. For example, MuSiC is not cited.
- Make sure all labels in all figures are readable. For example, Supplementary Fig 2 axes labels hard to read.
- Figure 6A would greatly benefit if the pathotype were ordered in each panel, in same order across panels, to be able to compare groups visually, and Figure 6B needs y axes labels (Cell freq either percentage, cell number, etc for each method).
- FACs not defined when first introduced, and a reference is needed to validate that sentence.
- Please make sure every statement made throughout manuscript, introduction and discussion particularly, is backed-up with at least one reference (for example: in discussion the authors say deconv. methods "fail outright"). If the authors are referribng to DWLS's failure in the current study, that was a cell number issue from the authors and not a DWLS failure.

This is not a cell number issue. Even if clusters with cell numbers <10 are removed, DWLS still reproducibly fails in the following circumstances: i) datasets/samples where some cell subclasses are absent (see reply 12 above and **Main Fig. 4c and Supplementary Fig. 19**); ii) certain types of added noise especially 'shift' noise; but most importantly, iii) real-world RNA-Seq e.g. synovium. We have provided the synovium RNA-Seq matrix for users to check this.

- NGD acronym is defined multiple times.

Text consistency has been checked.
Sentences have been checked.
References have been checked.
Figures have been modified so that they are readable.
**Supplementary Fig. 2**: the whole figure has been enlarged to make the font more readable.

An alternative version of **Fig. 6A** where pathotype is grouped and individuals are in the same order across deconvolution methods is depicted in **Supplementary Fig. 25c**.
Y axis labels for each method have been added for **Fig. 6b**.
FACs has now been defined when first introduced and a suitable reference has been added.
We have fixed the text to reduce the number of times NGD is defined.

(Remarks on code availability)

The above link is to the R package page, not the code. The manuscript has the link: https://github.com/myles-lewis/cellGeometry , which does contain the code for the package with a README with instructions on how to use it. however, this repository doesn't have the instructions to replicate the study results, even if the method's code is there.

We have provided the full source code for replication of the analyses and figures shown in the paper at https://github.com/EMR-bioinformatics/cellGeometry_manuscript/
Also see reply to Reviewer 1.

## REVIEWER COMMENTS

We thank all three reviewers for their helpful and constructive comments.

Reviewer #1 (Remarks to the Author):

I thank the authors for their substantive revision. I am satisfied with the responses to the majority of my points. Two issues remain where I believe the wording needs adjustment in the abstract:

1. Framing of NGD as a novel regularisation - The authors now show explicitly that NGD reduces to a constrained generalized Tikhonov problem with a heuristic, non-negativity-driven choice of the regularisation weight vector w rather than a CV-tuned scalar λ. This is a legitimate and useful variant, and I appreciate the added derivation. However, the abstract, introduction, and discussion still describe NGD as "a novel form of non-negative matrix regularisation" and as a "new paradigm". Given the derivation now in the Methods, I would ask the authors to align the framing with the math: position NGD as a constrained generalized Tikhonov method with a novel heuristic for the regularisation weights (driven by non-negativity of cell counts rather than cross-validation), rather than as a distinct paradigm. The scientific contribution is not diminished by this rewording, if anything, it is clarified.

We have reworded the text as suggested.

2. Generalisability claims given the absence of cross-platform validation - I accept the authors' argument that truly matched cross-platform (same-cells). However, the abstract still states cellGeometry "is robust to biases introduced by different sequencing chemistries", and the discussion generalises from pseudo-bulk simulations plus one synovium real-data validation. A reference-A to bulk-B design across independent cohorts/chemistries is not impossible and remains the cleanest test of the generality claim. At minimum, I would ask the authors to soften the chemistry-robustness language in the abstract and discussion to reflect that robustness has been demonstrated primarily through simulated noise models (log noise, shift noise) rather than empirical cross-platform data, and to explicitly acknowledge this as a limitation.

We have reworded the Abstract as suggested and made the limitations more explicit in the Discussion.

Reviewer #1 (Remarks on code availability):

I didn't test the code.

Reviewer #2 (Remarks to the Author):

Breakdown of my previous concerns:

Major comments:

3.1 Estimator / conditioning / NNLS comparison: largely addressed.
3.2 Domain shift as a primary benchmark: only partially addressed, but the response is reasonable.

3.3 Calibration-sensitive validation: mostly addressed, but incompletely. My request for interval calibration, especially nominal 90/95% coverage and stratification by cell-type similarity, was not addressed.

We have added confint() function to the package which allows users to calculate confidence intervals on fitted deconvolution models with variable alpha level (default 95%). We have also added a max_similarity() function, so that users can see which cell types have highest similarity to

other cell types. Using these 2 new functions, users can generate calibration intervals for their results and compare these against cell-type similarity. Both functions are fully documented and have been tested across the datasets used in the paper. In addition, the function diagnose() performs pairwise comparisons of cosine similarity between cell subclasses and produces a summarised printout and tables of the most similar pairs of cell subclasses.

*3.4 Residual layer as a first-class product: partially addressed. The residual workflow is now clearer and better supported, although it is not fully developed into the broader framework I had suggested.*
*3.5 Pathotype thresholds / stability: adequately addressed.*

*Minor comments: mostly addressed.*

*Overall: The revision has addressed most of my major concerns and has materially improved the manuscript. The authors have strengthened the statistical formulation of the method, added additional baseline and calibration analyses, and made the pathotype-thresholding procedure more data driven and defensible. One major concern remains only partially addressed, but there is no longer any issue that precludes publication, and I would support acceptance.*

*Reviewer #2 (Remarks on code availability):*

*I checked the public CRAN release of the package and verified from the package documentation that the main code-facing additions described in the revision are now present, including expanded deconvolution options, residual diagnostics, SE extraction, conditioning diagnostics, and signature-merging utilities. I did not independently rerun the full manuscript benchmarking pipeline, so this should be taken as confirmation of public implementation and documentation rather than a full reproducibility audit. The code availability is therefore substantially improved and, in my view, adequate for publication.*

*Reviewer #3 (Remarks to the Author):*

*1.We are specifically referring to merging of 2 distinct scRNA-Seq datasets. While this is possible manually, it requires aligning the rows of genes and binding columns of the 2 raw SC count matrices together, then concatenating the SC metadata, and adding a column for batch. However, that compute process is not trivial when the count matrix and metadata run into 30 GB file sizes, where the finalised merged objects need to be saved to disc. MuSiC has no way to specify multiple scRNA-Seq dataset objects as input. In our simulation of the brain dataset the simulated pseudo-bulk is generated by sampling from both the neuronal and non-neuronal SC datasets and then summing the resulting bulk gene expression. We show that analysis of the neuronal and non-neuronal datasets by MuSiC is i) extremely time consuming and ii) inaccurate. cellGeometry takes a different view. It merges only the signature matrices, not the raw single-cell count matrices. This is considerably more convenient for users and avoids manipulations of massive on-disc matrices. If the signature matrix may have a slightly different scale (this varies depending on number of cycles of PCR etc during library prep), the distributions of the signature matrices are aligned using QQ mapping before merging. With the brain atlas datasets, QQ mapping is not required as the 2 SC datasets were generated by the same lab using the same scRNA-Seq methodology. In this situation cellGeometry is able to deconvolute simulated bulk brain RNA-Seq containing both neuronal and non-neuronal cells without degradation in accuracy.*

*The authors' response does not address my concern. A valid method comparison requires each tool to be evaluated within its intended use case. MuSiC was not designed for atlas-scale, multi-dataset deconvolution, and benchmarking it in that setting is circular: the experimental framework was designed around cellGeometry's strengths. If dataset size is the barrier to running MuSiC appropriately, the solution is to downscale the dataset to a size where MuSiC can operate as*

*intended, then perform the comparison with cellGeometry. If cellGeometry still outperforms in accuracy under those conditions, that is a legitimate result worth reporting. The authors should either perform the comparison on an appropriately scoped dataset, or remove the superiority claim and reframe cellGeometry's atlas-scale capability as a novel extension rather than a benchmarked advantage.*

This is a valid point. We have re-run the analyses of the 2 largest, atlas-scale datasets (Tabula & brain) with MuSiC applied to downscaled scRNA-Seq datasets, using the same downscaling as was used for DWLS and LinDeconSeq. Although this rendered modest improvements in the speed of MuSiC, it did not improve the accuracy of deconvolution. We have added the results of MuSiC with the downscaled datasets to Supplementary Table 1.

*2. cellGeometry removes cell subclasses with <10 cells in the scRNA-Seq by default since we believe there is insufficient information if fewer than 10 cells are present. This cutoff can be controlled by users through a parameter setting. The authors of DWLS state that one of the rationales of DWLS was to overcome rare cell population deconvolution, but they do not specify how rare. Both MuSiC, DWLS and LinDeconSeq give no specific instructions for removing cell subclasses below a particular threshold. MuSiC2 was designed with 2 bulk tissue datasets in mind (control vs disease) to be able to handle the situation where some cell subclasses might be rare and only present in one bulk dataset. However, we could not find a way to use this method as we did not have matching control vs disease bulk datasets and had no easy way to simulate this. MuSiC2 removes cell subclasses where the mean proportion of cells across all samples in the bulk data is < 10% of the cells as the default (prop_r = 0.1). This means it is mainly designed only to detect much more common cells and will remove rare cells in bulk by default. MuSiC1 & 2 do not specify a cutoff for the number of cells in the scRNA-Seq dataset. We re-ran MuSiC, LinDeconSeq and DWLS for the deconvolution of real-world whole blood bulk RNA-seq whilst removing single-cell clusters with <10 cells in the reference. This hardly altered their deconvolution results (Table R1 below) and the correlation between predicted lymphocyte percentages and lymphocyte Coulter Counter measurements did not improve (Fig. R7 below). In the AMP synovium dataset, the minimum number of cells per subclass was 48. We considered all of these subclasses to be important and functional, as described in the AMP paper (Zhang F 2019, Nature immunology, PMID: 31061532). As we believe from earlier histological studies that we are likely to find all 14 of these cell types within inflamed rheumatoid arthritis synovial tissue (albeit not in all patients all of the time), we did not remove any of these subclasses. We provide the bulk synovium RNA-Seq dataset for users to test out DWLS for themselves.*

The authors' response does not address my concern, which is still related to the one above. Comparing methods in a way they are not intended to be used, then claiming cellGeometry superiority in accuracy etc. If the authors want to keep their current methodology in benchmarking (i.e., atlas-level scales of data etc.), please choose methods that are intended to be used at this level of data. Bisque and InstaPrism are 2 options that can serve in this framework when comparing performance at atlas-level scale.

We have tested Bisque and InstaPrism on the 2 atlas-level datasets. Full results have been added to Supplementary Table 1 and correlation plots are shown in Supplementary Fig. 9-11. Neither of these packages were as accurate as cellGeometry or DWLS on the Tabula Sapiens datasets. Both Bisque and InstaPrism performed poorly on the brain dataset in comparison to cellGeometry.

Notably, although Bisque has been tested on atlas-scale datasets, its software package is **not** capable of handling the large-scale dataset formats such as the DelayedArray matrix format and h5ad files. Like DWLS and LinDeconSeq it is also limited to $2^{31}$ matrix elements. Thus, in order to test Bisque, sc/snRNA-Seq matrices had to be downsampled.

*3-To overcome the matrix limit of $2^{31}$ elements, we restricted the maximum number of cells to a fixed amount and sampled for the cell subclasses randomly to stay within the $2^{31}$ limit. This reduced each of the 1-3 million cell scRNA-Seq datasets to around 20-30,000 cells. The subsetted Human Brain Cell Atlas and Tabula Sapiens datasets were used to assess deconvolution by*

*DWLS and LinDeconSeq. In spite of subsetting the cells, LinDeconSeq was unable to deconvolute both Human Brain Cell Atlas (neuron) and Tabula Sapiens. It failed at the signature generation step. Full details and a reprex script have been provided on the Github repo: https://github.com/EMRbioinformatics/cellGeometry_manuscript/tree/main/02_Stimulated_benchmarking/No_noise/LinDeconSeq_ reprex Even with the scRNA-Seq subset to 20-30,000 cells, DWLS took 54 hours to generate the gene signature for Tabula Sapiens. Although DWLS had high accuracy for deconvoluting Tabula Sapiens, DWLS could only consider neuron and non-neuronal cells separately and so did not perform as well (main Fig. 1c [revised]). Human Brain Cell Atlas non-neuronal simulated data was deconvoluted using DWLS and LinDeconSeq where both did not perform well and were significantly worse than cellGeometry (Supplementary Figure 17).*

I understand that subsetting to 20-30,000 also caused the comparison methods to break down mathematically. This is expected; a scRNA-seq reference in practice would very rarely have more than 2,000 per cell type. If the programming language is the issue, most methods have a Python implementation, or other methods can be tested. DWLS is notoriously noted as a slow method not meant for large datasets in benchmarking papers (https://www.nature.com/articles/s41467-020-19015-1). To highlight cellGeometry's ability to deconvolve 1. Fast and 2. Using atlas level dataset, it would be much better to compare to methods that are meant for this, like InstaPrism, Bisque. This is related to my comments above; appropriate comparison would strengthen the manuscript.

See comment above. We have compared cellGeometry to InstaPrism and Bisque on the atlas-scale datasets.

*4. The Human Brain Cell Atlas is single-nucleus RNA-seq, whereas the other datasets (AMP rheumatoid arthritis synovium, Cell Typist and Tabula Sapiens) are single cell. We have clarified this in the text.*

I believe this to be a larger concern than stated. There are methods specifically designed to use single-nucleus RNA-seq references as reference. Is this also the intention with cellGeometry? Increasing research has shown single-cell and single-nucleus are not interchangeable as references. The manuscript would benefit from explicitly experimentally showing single-nucleus is an effective reference for this cellGeomtry, or comparing the single-nucleus performance to methods that also use single-nucleus rather than methods that use single-cell.

We do not have access to a study with the three necessary, matching datasets which would be needed to render this comparison, and we feel that it is beyond the scope of the current manuscript. To perform this comparison properly, matched samples would need to undergo both single-cell and single-nucleus RNA-Seq as well as bulk RNA-Seq. Then deconvolution of the bulk could be compared against actual cell number results in scRNA-Seq vs snRNA-Seq to see whether certain cell populations were accurately deconvoluted.

However, one study suggested that snRNA-Seq reference performs well in comparison to scRNA-Seq for deconvolution of brain (PMID: 35292647), although their conclusions are based partly on simulations of pseudo-bulk data, as this study did not have access to bulk and sc/snRNA-Seq performed on the same samples. The methods cited by this study (CIBERSORT, MuSiC) were not altered specifically for snRNA-Seq vs scRNA-Seq deconvolution.

We have added text to discuss these points in a paragraph in the Discussion which discusses limitations of the present study.

*5. Using the genes that were filtered out by cellGeometry for the deconvolution of whole blood RNA-seq from the PEAC clinical study using the Cell Typist blood dataset (as described in main Fig. 5a-c), we have removed them from the DWLS signature and from the Cell Typist blood reference for MuSiC. MuSiC is a one-step deconvolution process so the signature cannot be directly intercepted and adjusted accordingly. It was noted that LinDeconSeq gene signature already did not contain the genes filtered out by cellGeometry. 16 With these adjustments, whole blood RNA-seq deconvolution was rerun for MuSiC and DWLS (Fig. R8 ab below) where we generate same comparisons as main Fig. 5e & g (for ease of comparison Fig. R8 c-d below). The correlation of lymphocyte percentages with Coulter counter measurements improved slightly, but*

*was still not as good as cellGeometry (r = 0.53, p = 3.3e-05). We have noted in the manuscript text that gene filtering of the gene signatures in other deconvolution methods was performed, but only partially improved accuracy. Both MuSiC and DWLS still overestimated monocytes and underestimated T cells.*

The authors have addressed my comment.

*6. These were cell subclass percentages rather than fractional proportions which add up to 1. The legend has been amended to clarify. The correlation plots for Tabula Sapiens and the Human Brain Cell Atlas simulated data have been consolidated into single plots for each deconvolution method (Supplementary Fig. 9-11).*

The authors have addressed my comment.

*7. This is addressed in Fig. 6c where the MuSiC estimated T cells do not correlate with CD3 histology.*

Thank you for clarifying. However, those values appear to be very close. Is there a statistically significant difference?

Correlation between cellGeometry estimated T cells and histology scores was r = 0.50, P = 2.4e-12, whereas for MuSiC the correlation between estimated T cells and histology scores was r = -0.086, P = 0.26. The difference between these correlations is statistically significant at P = 4.50e-09 (Z = 5.86, using Fisher's r to Z transform).

*8. There are 2 main reasons:*
*i) Since the RNA data is on count scale, Gaussian noise barely touches genes with high expression e.g. whose counts run into 10^5 or more. As these genes are untouched and often more highly leveraged in deconvolution methods, highly expressed genes provide full information for the algorithm.*
*ii) Gaussian noise does not change Rsq unless the noise is very, very large because the noise is unbiased. So on a population level most methods use many genes (equivalent to large n) and if each gene a b MuSiC DWLS MuSiC DWLS c d Rerun with gene filtering Original 17 is affected equally by noise, then if the deconvolution method is BLUE and so based on averaging inference from many genes it is not affected much by Gaussian noise. Noise methods with intrinsic bias will more heavily affect these systems. Non-Gaussian noise (log, sqrt) will be upwardly biased once converted back to count scale. Because shift_noise selects genes at random, this causes bias in whole genes. If multiple genes which are cell-specific are shifted this will alter the deconvolution in a biased, nonsymmetric way, so methods are more sensitive to this.*
I want to tank the authors for their explanation, but have concerns about all arguments presented.
i) This is inconsistent with how these methods operate. MuSiC explicitly downweights broadly highly expressed genes, and DWLS is built around marker gene selection. The genes driving deconvolution are cell-type-specific markers, not globally highly expressed genes, so this argument should be revised or removed.
Regarding point (ii) and the figure more broadly: While the unbiasedness argument is statistically sound, an equally plausible explanation for the flat $R^2$ is simply that the Gaussian noise levels tested (SD=100–500) are trivially small relative to the dynamic range of the data, which from Supp. Fig. 18a clearly spans up to $10^8$ counts. The two explanations cannot be distinguished from the data as presented. The authors should test substantially larger Gaussian noise levels to determine whether the flat $R^2$ reflects true robustness or merely insufficient noise magnitude. As currently presented, this analysis does not convincingly support either conclusion.

We have tested substantially larger Gaussian noise levels that are 10 times the original noise levels (SD = 100-500 to SD = 1000-5000). Main **Fig. 4b** and **Supplementary Fig. 18a-b** have been updated. **Fig. R1** (below) shows that deconvolution accuracy of both cellGeometry and DWLS worsens as Gaussian noise increases – it is not flat. Both MuSiC and LinDeconSeq already performed poorly in comparison to cellGeometry and DWLS, and show less change with the

increase in Gaussian noise. Our interpretation is that MuSiC and LinDeconSeq may rely on a few very highly expressed genes which are much less affected by Gaussian noise. This, plus the fact these two methods are much less accurate to start with, may be why their graphs are flatter than those of cellGeometry and DWLS.

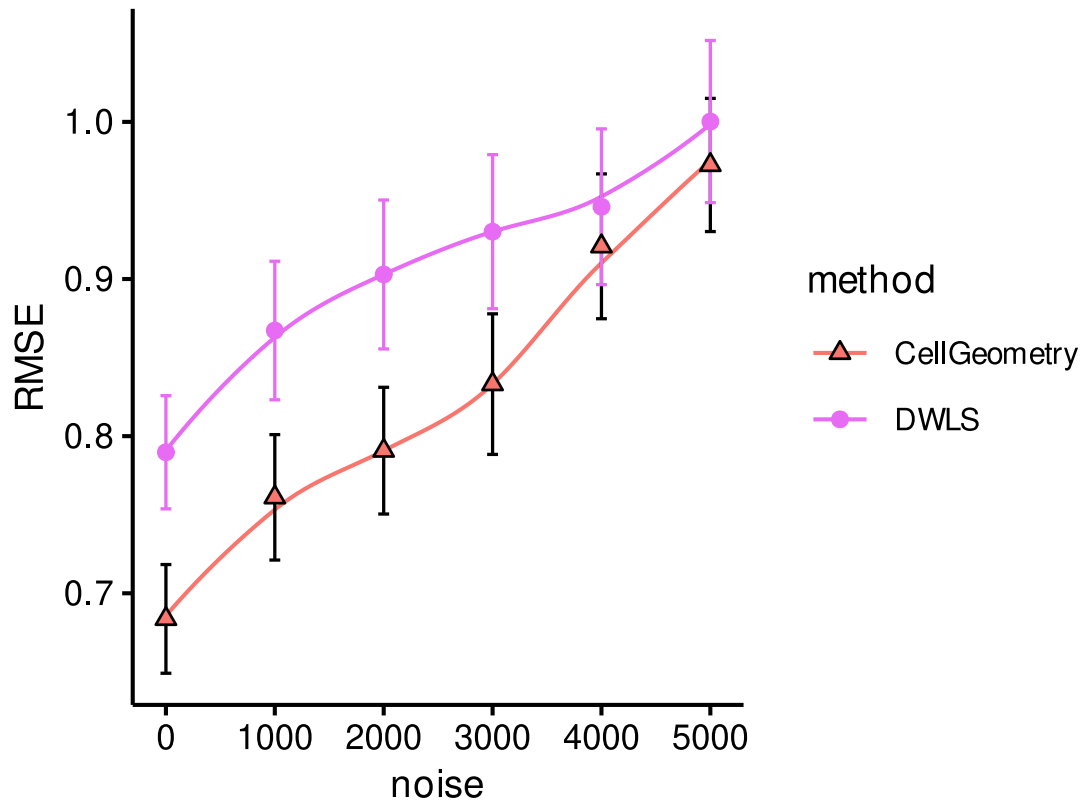


**Fig. R1.** RMSE of cell subclass percentages from the deconvolution of the simulated pseudo-bulk data from RA synovium scRNA-Seq with addition of varying amounts of Gaussian noise

Reviewer #3 (Remarks on code availability):

The code is present in the repository, but no instructions on replicating the experiments are included.

Detailed instructions for users new to the package are available on CRAN through two vignettes. The first vignette gives instructions for installation of the package and necessary dependencies as well as leading first time users through basic analysis of the Cell Typist dataset via creation of a simulated pseudo-bulk dataset. The $2^{nd}$ vignette gives detailed instructions for creation of merged simulated pseudo-bulk from the 2 brain atlas scRNA-Seq data files and walks users through analysis of this dataset. We have added links in the code reproducibility Github repo pointing users to the vignettes in the main CRAN repository webpage. This will make it easier for users to get started with the reproducibility code provided, thus allowing users to reproduce all aspects of the results in his manuscript.

Reviewer #2 (Remarks to the Author):

Assessment of whether Reviewer #3's remaining concerns have been addressed:

I reviewed the revised manuscript, rebuttal, and supplementary material with the specific goal of assessing whether the remaining concerns raised by Reviewer #3 have been adequately addressed. Overall, I think the authors have now addressed the main substance of Reviewer #3's concerns. The original concern about fairness in the atlas-scale benchmarking was valid, but the revised manuscript now includes the key missing controls: downsampled MuSiC, Bisque, and InstaPrism. These additions make the benchmarking much more defensible. I do not see a remaining Reviewer #3 issue that should block publication. I would still ask for a few small wording and correctness edits.

1. Atlas-scale benchmarking and MuSiC comparison:

Reviewer #3's main concern was that the original comparison was not fully fair: cellGeometry was being tested in a setting that used its strengths, especially very large references and merging separate references, while MuSiC was not designed for that exact use case.

The authors have now addressed this in a reasonable way. They reran MuSiC on downsampled Tabula Sapiens and Human Brain Cell Atlas references, using the same broad downsampling strategy used for other methods. This was the correct control to add. The downsampled MuSiC results do not close the accuracy gap, so the original conclusion is now better supported.

The authors also added Bisque and InstaPrism, which directly addresses Reviewer #3's request to include methods more appropriate for atlas-scale or large-reference settings. These comparisons are now included in the supplementary figures and Supplementary Table 1. This substantially improves the fairness of the benchmark.

My only remaining issue is the wording around the Human Brain Cell Atlas result. In that analysis, cellGeometry is not only being compared as an estimator; it is also being tested for its ability to merge neuron and non-neuronal reference signatures. That is a real and useful feature. But it is not a perfectly apples-to-apples estimator comparison, because the other methods are not set up to merge signatures in the same way. I would ask the authors to frame this result more explicitly as a demonstration of cellGeometry's merged-reference capability, rather than only as a pure accuracy benchmark.

Note: this is a wording issue, not a request for new analysis.

We have amended the manuscript (p.9-10) where we write:

This is demonstrated through the Human Brain Cell Atlas which contains neurons and non-neuronal cells in separate files. Simulated pseudo-bulk samples containing random mixtures of neurons and non-neuronal cells were generated (Fig. 3a-c). MuSiC, DWLS and LinDeconSeq do not have this feature and so the predicted percentages can only consider neuron and non-neuronal separately, which is poorly representative of the actual percentages of the simulated data. Consequently, the reference merging feature of cellGeometry contributes to its substantially greater accuracy compared to other methods (Fig. 3d-e, Supplementary Fig. 16-17).

Here we make it explicitly clear that cellGeometry's better results are explained by the merged reference capability and explain why the other methods perform less well.

2. Comparator methods for atlas-level data: This concern is addressed.

Reviewer #3 specifically suggested Bisque and InstaPrism. The authors added both. The supplementary table and plots show that these methods did not outperform cellGeometry on the atlas-scale benchmarks. This is the right response.

One small wording point: the phrase “purportedly designed for large-scale sc/snRNA-Seq studies” sounds a little dismissive. I would suggest replacing it with something more neutral, such as “methods applied to large-scale sc/snRNA-Seq studies” or “methods intended for large-scale reference-based deconvolution.” The authors do not need to weaken the result, but the wording should stay neutral.

We have revised the wording as recommended.

3. Downsampling and intended use of methods: This is mostly addressed.

The authors now explain how large references were downsampled for methods that could not handle the full matrix size. They also tested downsampling for MuSiC. That addresses the main fairness issue.

I would still keep two points separate in the manuscript: 1st, cellGeometry scales better computationally; 2nd, cellGeometry is more accurate in these benchmarks. These are related, but they are not identical claims. The revised manuscript mostly makes this clear, but the brain atlas section still risks mixing “feature advantage” with “estimator accuracy.” This can be fixed by wording.

See comment #1 above. We have amended the brain atlas results paragraph for clarity re cellGeometry’s “feature advantage”.

4. Single-nucleus RNA-seq reference issue: This is the only Reviewer #3 concern that is not fully experimentally resolved. But I think the authors’ response is acceptable.

Reviewer #3 correctly pointed out that the Human Brain Cell Atlas is single-nucleus RNA-seq, and that scRNA-seq and snRNA-seq references are not interchangeable. The authors now state in the main text that the benchmark includes three scRNA-seq datasets and one snRNA-seq dataset. They also added limitation language explaining that a proper test would require matched scRNA-seq, snRNA-seq, and bulk RNA-seq from the same samples.

I agree that such a matched experiment is beyond the scope of this revision. I would not block the paper on this point. However, the manuscript needs to be careful and consistent. The current evidence supports that cellGeometry can be applied to the Human Brain Cell Atlas snRNA-seq reference in this simulation setup. It does not prove that snRNA-seq references are generally validated for real bulk deconvolution across tissues.

I would ask for small wording corrections wherever the Human Brain Cell Atlas is included. Some text still uses “scRNA-seq datasets” generically when the Human Brain Cell Atlas is part of the comparison. This should be changed to “sc/snRNA-seq datasets” or “single-cell/single-nucleus reference datasets.” This applies especially to the abstract, figure legends, and Supplementary Table 1 caption. This is a correctness issue.

We have updated the text as requested.

5. Gene filtering / residual gene removal: This concern is addressed.

Reviewer #3 asked whether cellGeometry’s advantage could come from its residual gene filtering rather than the deconvolution method itself. The authors tested removing the same outlier genes from the inputs/signatures used by other methods and found only small improvements. This is an adequate response.

6. Percentages versus proportions: This is addressed.

The revised legends and supplementary plots now clarify that the metrics are based on predicted versus true cell percentages/subclass percentages. I have no further concern here.

7. Statistical comparison of histology correlations: This is mostly addressed.

Reviewer #3 asked whether the apparent difference between cellGeometry and MuSiC in the T-cell/CD3 histology comparison was statistically significant. The authors report in the rebuttal that the difference is significant by Fisher r-to-z test.

That answers the question. However, I think the Fisher r-to-z result should be added to the manuscript or supplement, not only left in the rebuttal. This is a small edit, but it would make the statistical clarification visible to readers.

We have added the Fisher Z statistics to the manuscript.

8. Gaussian noise analysis: This concern is addressed.

Reviewer #3’s objection was logical: the original Gaussian noise levels may have been too small relative to the dynamic range of the count data, so the flat performance curve might not mean true robustness. The authors responded by testing substantially larger Gaussian noise levels and updating the main and supplementary figures. The revised plots now show performance changes as Gaussian noise increases.

That resolves the concern.

I would only avoid overexplaining why MuSiC or LinDeconSeq curves are flatter unless that mechanism is directly tested. The revised manuscript does not need much speculation here. It is enough to say that larger Gaussian noise levels were tested and that the main conclusions are unchanged.

9. Code availability and reproducibility instructions: This is adequately addressed for publication.

The authors now point users to CRAN vignettes and provide benchmarking/figure-generation scripts. I did not rerun the full benchmarking pipeline, so this is not a full reproducibility audit. But the code/instruction issue raised by Reviewer #3 has been improved enough.

Overall assessment

I think Reviewer #3’s main concerns have been substantively addressed. The most important fixes were the downsampled MuSiC analysis, the addition of Bisque and InstaPrism, the expanded Gaussian noise benchmark, the gene-filtering control for other methods, and clearer code/vignette information.

The only point that remains partially addressed is the snRNA-seq issue. The authors did not add matched scRNA/snRNA/bulk validation, but they now acknowledge this limitation. I think that is acceptable for this manuscript, as long as the claims are cautious and the terminology

is corrected.

I would support acceptance after minor wording/correctness edits:

1. Frame the Human Brain Cell Atlas result as a merged-reference capability result, not a purely apples-to-apples estimator comparison.
2. Use “sc/snRNA-seq” or “single-cell/single-nucleus reference datasets” consistently wherever the Human Brain Cell Atlas is included, including the abstract, figure legends, and supplementary table captions.
3. Add the Fisher r-to-z comparison for the CD3/T-cell correlation difference to the manuscript or supplement.
4. Remove or soften slightly dismissive wording such as “purportedly designed” when describing comparator methods.

These are minor edits. I do not think any remaining Reviewer #3 concern requires further major analysis.

We have made amendments to fix these comments 1-4 as described above.

Reviewer #2 (Remarks on code availability):

This is adequately addressed for publication.
The authors now point users to CRAN vignettes and provide benchmarking/figure-generation scripts. I did not rerun the full benchmarking pipeline, so this is not a full reproducibility audit. But the code/instruction issue raised by Reviewer #3 has been improved enough.
